# Acceptor-Donor-Acceptor *π*-Stacking Boosts Intramolecular Through-Space Charge Transfer towards Efficient Red TADF and High-Performance OLEDs

**DOI:** 10.34133/2022/9892802

**Published:** 2022-06-24

**Authors:** Chenglin Jiang, Jingsheng Miao, Danwen Zhang, Zhenhua Wen, Chuluo Yang, Kai Li

**Affiliations:** ^1^Shenzhen Key Laboratory of New Information Display and Storage Materials, College of Materials Science and Engineering, Shenzhen University, Shenzhen 518055, China; ^2^College of Physics and Optoelectronic Engineering, Shenzhen University, Shenzhen 518060, China

## Abstract

Organic push-pull systems featuring through-space charge transfer (TSCT) excited states have been disclosed to be capable of exhibiting thermally activated delayed fluorescence (TADF), but to realize high-efficiency long-wavelength emission still remains a challenge. Herein, we report a series of strongly emissive orange-red and red TSCT-TADF emitters having (quasi)planar and rigid donor and acceptor segments which are placed in close proximity and orientated in a cofacial manner. Emission maxima (*λ*_em_) of 594−599 nm with photoluminescence quantum yields (PLQYs) of up to 91% and delayed fluorescence lifetimes of down to 4.9 *μ*s have been achieved for new acceptor-donor-acceptor (A-D-A) molecules in doped thin films. The presence of multiple acceptors and the strong intramolecular *π*-stacking interactions have been unveiled to be crucial for the efficient low-energy TSCT-TADF emissions. Organic light-emitting diodes (OLEDs) based on the new A-D-A emitters demonstrated electroluminescence with maximum external quantum efficiencies (EQEs) of up to 23.2% for the red TSCT-TADF emitters. An EQE of 18.9% at the brightness of 1000 cd m^−2^ represents one of the highest values for red TADF OLEDs. This work demonstrates a modular approach for developing high-performance red TADF emitters through engineering through-space interactions, and it may also provide implications to the design of TADF emitter with other colours.

## 1. Introduction

The past decade has witnessed a booming research interest in seeking luminescent purely organic push-pull molecules that can harvest triplet excitons for organic light-emitting diode (OLED) applications owing to their advantages over noble metal complexes in terms of low cost. Rather than direct radiative decay of the lowest lying triplet excited state (T_1_) to give phosphorescence, the upconversion of T_1_ excitons to singlet ones (S_1_) which subsequently radiate to give thermally activated delayed fluorescence (TADF) has been devised for organic molecules to circumvent their very slow phosphorescent decay processes [1, 2]. It can be seen that a key to efficient TADF is a fast T_1_→S_1_ reverse intersystem crossing (RISC) process which is executed by an electron spin-flip. According to the Fermi golden rule under the Condon approximation, RISC rate correlates proportionally with spin-orbit coupling matrix element (SOCME) between the coupling singlet and triplet excited states and inversely with their energy difference (Δ*E*_ST_). Taking into account of the very small SOCME for purely organic molecules devoid of a heavy atom, a rapid RISC takes place only when the coupling singlet and triplet excited states are close in energy, that is, a trifling Δ*E*_ST_. To this end, it has been a consensus to design molecules with spatially separated highest occupied molecular orbital (HOMO) and lowest unoccupied molecular orbital (LUMO) which can effectively minimize the exchange interaction. Following this principle, efficient TADF emissions with widely tunable excited state properties including energies and lifetimes have been realized through connecting an electron donor (D) and an acceptor (A) in a twisted manner [3–5].

Alternatively, construction of molecular scaffolds featuring intramolecular through-space charge transfer (TSCT) excited states as opposed to through-bond charge transfer (TBCT) between the D and A offers another molecular design of TADF emitters on account of their intrinsically small Δ*E*_ST_ values [6–21], whereas early efforts to develop TSCT-TADF molecules have been frustrated by the weak through-space electronic coupling within the D-A pair and the presence of abundant intramolecular motions [17–21]. In this context, significant enhancements in photo- and electroluminescence efficiencies have recently been achieved for TSCT-TADF small molecules, polymers, and dendrimers (Figure S1) [22–33]. Remarkably, Kaji and coworkers reported a significantly boosted RISC rate for a triptycene-supported molecule by virtue of a vibronically coupled spin-orbit coupling (SOC) effect [30]. The space-confining effect has been exploited for greatly suppressing the nonradiative decay of TSCT excited states [31–33]. However, high-efficiency TSCT-TADF emissions have been mainly limited to the blue-to-green regime with only few reports on relatively weak red emissions [25, 28]. Although various D-A combinations have been developed for widely tuning the colours of TBCT-TADF emitters, they may not be well suited for the design of TSCT-TADF emitters if their geometric structures do not favour through-space interactions [34]. In particular, the suppression of nonradiative decay is essential for efficient red emissions to offset the energy gap law [35, 36]. Therefore, a molecular design that allows for simultaneous manipulations of all these parameters has become an imperative demand.

**Figure 1:**
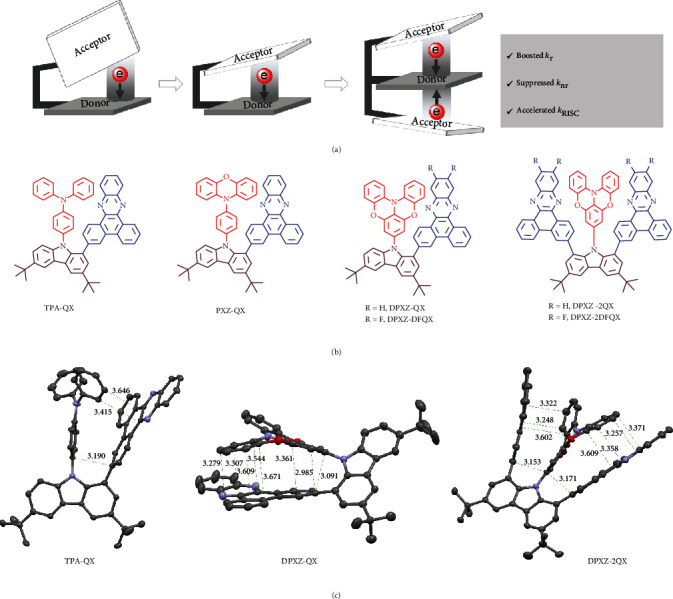
Design principle and structures of compounds in this study. (a) Illustration of the molecular design of A-D-A-type TSCT emitters with a sandwich configuration. (b) Chemical structures of *TPA-QX*, *PXZ-QX*, *DPXZ-QX*, *DPXZ-DFQX*, *DPXZ-2QX*, and *DPXZ-2DFQX*. (c) Perspective views of the single crystal structures of *TPA-QX*, *DPXZ-QX*, and *DPXZ-2QX* with key short C-*π*/*π*-*π* distances indicated.

The cofacial donor-acceptor orientation has proven crucial for regulating the electronic communications and thus the excited state dynamics of push-pull dyads, which is an important topic in the studies of charge-separated states [37–39]. However, its implications on TADF properties have been scarcely explored. We recently demonstrated highly efficient green TADF and phosphorescence emissions by confining quasiplanar motifs to have a face-to-face orientation [40, 41]. We envisioned that this molecular design leveraging on strong intramolecular *π*-stacking interactions would be able to break the efficiency limit on long wavelength TSCT-TADF emissions, as opposed to an edge-to-face orientation. Herein, we designed four molecules, *DPXZ-QX*, *DPXZ-DFQX*, *DPXZ-2QX*, and *DPXZ-2DFQX* using planar dibenzo[a,c]phenazine (QX) and its fluorinated derivative (DFQX) as the acceptors (Figure 1) [42–49]. The quasiplanar O-bridged triphenylamine (DPXZ) was used as the donor [40, 41, 50]. For comparison, triphenylamine (TPA) and phenoxazine (PXZ) were used as the donors to prepare the control compounds *TPA-QX* and *PXZ-QX*. Single-crystal X-ray diffraction studies revealed substantial face-to-face alignment of the donor and acceptor and thus strong *π*-*π* interactions in the four molecules containing DPXZ. These four emitters exhibit orange-red to red TSCT-TADF emissions with photoluminescence quantum yields (PLQYs) of 70−91% in doped films and have demonstrated OLEDs (*λ*_em_ = 588–617 nm) with maximum external quantum efficiency (EQE) over 23%. In stark contrast, TADF properties were not observed for *TPA-QX* and *PXZ-QX*, revealing the crucial role of face-to-face orientation in promoting electronic communications for TSCT-TADF emission.

## 2. Results

### 2.1. Molecular Design, Synthesis, and Structures

Geometries of the donor and acceptor and their relative orientation constitute the two key factors steering the strength of *π*-*π* interactions. As shown in Figure S1, the two planes at orthopositions of five- and six-membered rings are largely deviated from a parallel orientation. In these two cases, close contacts only exit between atoms near the bridge. Other prevailing bridges based on anthracene- and xanthene-type skeletons have been used for underpinning two planes in parallel [51]. However, only intramolecular *π*-*π* interactions between partial planes on the rigid anthracene can be found after significant torsion. Despite the shortening of distance between the anchorages in a bent geometry in xanthene, its flexibility is deleterious to emission efficiency [21]. In contrast, carbazole and fluorene derivatives can confine the two planes to be in proximity with a large degree of *π*-*π* overlap [31–34, 40, 41]. In addition, this kind of skeletons allows for a construction of sandwich-type molecular architecture in which intramolecular interactions can be further strengthened. More importantly, the presence of multiple donors/acceptors has proven capable of boosting RISC between states with different orbital configurations [52–56]. Illustration of the design concept from “edge-to-face” to sandwich-type “face-to-face” is shown in Figure 1(a), following which the emitters in this study have been developed (Figure 1(b)).

All target compounds were prepared by a three-step procedure (Supplementary Materials) and obtained as yellow-to-orange powders. Their structures were characterized by ^1^H and ^13^C NMR spectroscopy, high-resolution mass spectrometry, elemental analysis, and single-crystal X-ray diffractions. Thermogravimetric analysis (TGA) revealed high decomposition temperatures (*T*_d_ at a 5% weight loss) of 402−470°C for all the six compounds under Ar, among which the lowest thermal stability was observed for *TPA-QX* (Figure S2).

All single crystals of the present compounds were grown by slow evaporation of their solutions in mixed dichloromethane (DCM)/hexane. The crystal data are compiled in Tables S1–S3 (Supplementary Materials). As depicted in Figures 1(c) and S3, the QX and DFQX in all molecules have a flat geometry and are tilted with respect to the carbazole plane (torsion angles: 48−70°). The short distances (ca. 3.5 Å) between the donor and acceptor segments in *DPXZ-QX*, *DPXZ-DFQX*, *DPXZ-2QX*, and *DPXZ-2DFQX* signify strong *π*-*π* stacking interactions. Of note, the attractive force buckles the DPXZ to allow for a parallel orientation of the participating Ph ring relative to the QX plane. The force also leads to an antiparallel orientation of the two QX/DFQX planes in *DPXZ-2QX* and *DPXZ-2DFQX*, endowing them with a C_2_ symmetry. A pair of enantiomers are observed for the crystals containing DPXZ. Differently, there is no evident short *π*-*π* contact in *TPA-QX* and *PXZ-QX*. Instead, C-H⋯*π* interactions result from the edge-to-face alignment of the donor and acceptor.

**Figure 2:**
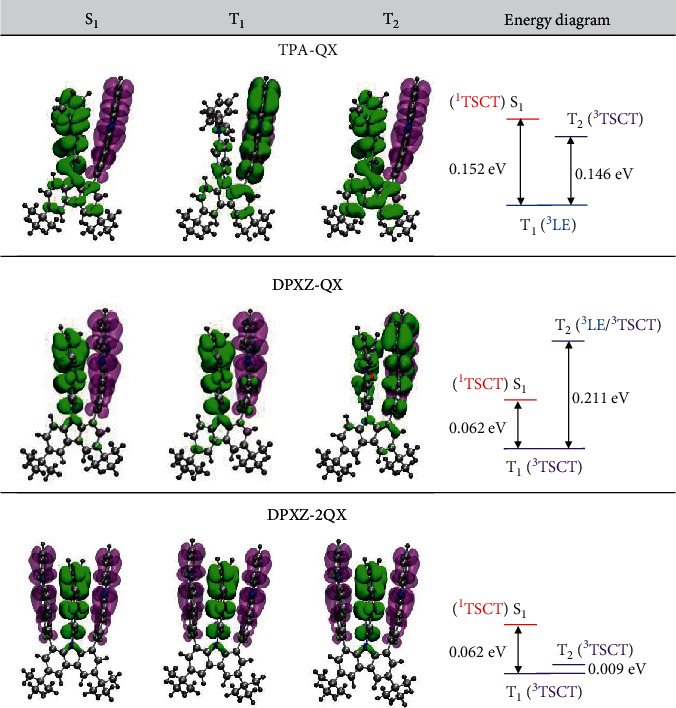
Theoretical simulations. NTOs of the S_1_, T_1_, and T_2_ states at optimized S_0_ structure (green: hole; purple: particle) and calculated energy level of each state for *TPA-QX*, *DPXZ-QX*, and *DPXZ-2QX*.

To validate the rational design in this work, the electronic structures of all molecules were studied through theoretical calculations using dispersion-corrected density functional theory (DFT) and time-dependent DFT (TDDFT) [57]. First, theoretical insights into the intramolecular *π*-*π* stacking interactions were gained by reduced gradient density (RGD) analysis of the optimized ground state (S_0_) structure [58]. The spikes within ±0.02 a.u. of the sign (*λ*_2_)*ρ* value reveal the presence of noncovalent interactions which are the strongest in the A-D-A structures (Figure S4). It can be clearly seen from the RDG isosurfaces that the noncovalent interactions are mainly confined to the space between the donor and acceptor in each molecule. As illustrated in Figure S5, the HOMO in the S_0_ geometry for each molecule is predominantly localized on the donor (TPA, PXZ, or DPXZ) with a very minor contribution from the nonbonding p orbital of the N(carbazoyl) atom. The LUMO is localized on the QX/DFQX moiety. Natural transition orbital (NTO) analyses of their S_1_ states show a TSCT nature (Figures 2 and S5). However, the T_1_ states of *TPA-QX* and *PXZ-QX* are dominated by a local-excitation (LE) character of QX, in contrast to the TSCT nature of T_1_ states for the others. This difference is caused by the destabilized ^1,3^TSCT states when TPA or PXZ is used, which also result in relatively larger Δ*E*_ST_ values. Therefore, an energy diagram that does not favour the TADF process is obtained for *TPA-QX* and *PXZ-QX*. Of interest, the presence of multiple acceptors imparts the sandwich-type emitters *DPXZ-2QX* and *DPXZ-2DFQX* dense excited states that should facilitate the RISC process as per the El-Sayed rule [52–56]. It is worth mentioning that the literature reports on engineering of multiple charge transfer excited states are majorly limited to D-A-D-type molecules with few exceptions of A-D-A congeners [59]. Importantly, the calculated oscillator strength (f) values of up to 10^−2^ are significantly high among the TSCT transitions, implying strong electronic coupling between HOMO and LUMO in *DPXZ-2QX* and *DPXZ-2DFQX*.

### 2.2. Photophysical Properties

**Figure 3:**
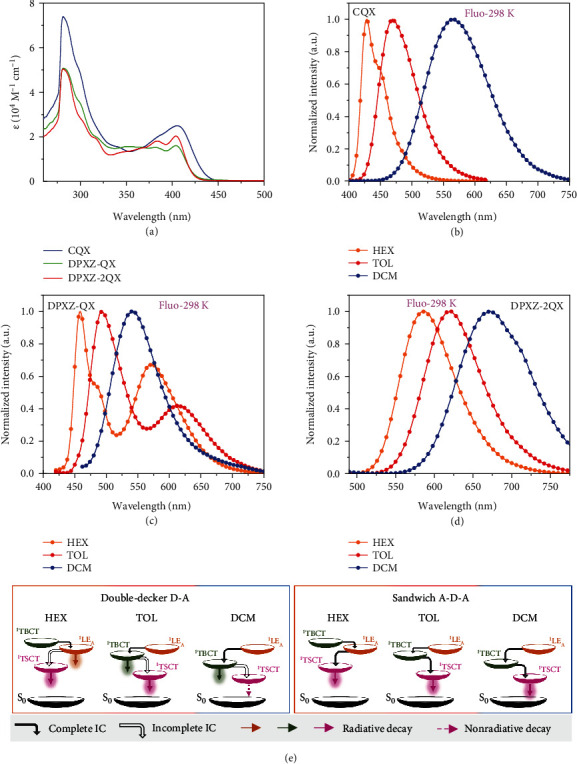
Photophysical properties and proposed plausible mechanisms in solutions. (a) UV-Vis absorption spectra of *CQX*, *DPXZ-QX*, and *DPXZ-2QX* in toluene (concentration: ~10^−5^ M). (b–d) Fluorescence spectra of (b) *CQX*, (c) *DPXZ-QX*, and (d) *DPXZ-2QX* in different solvents at 298 K. (e) Proposed state diagrams and decays of the multiple excited states for the double-decker and sandwich-type TSCT emitters dependent on the solvent polarity. The ISC and RISC processes involving triplet manifolds have been omitted for the sake of clarity.

The electronic spectra of all the cofacial D-A and A-D-A stacks were recorded in toluene at room temperature. The four precursors *CQX*, *CDFQX*, *C2QX*, and *C2DFQX*, which do not contain the D moiety (Supplementary Materials), were studied under the same conditions. As depicted in Figures 3(a) and S6, all of the compounds exhibit similar UV-Vis absorption spectral profiles. The intense absorptions in the region below 300 nm and in 340−410 nm are assigned to *π*-*π*∗ and charge transfer (carbazole→QX/DFQX) transitions, respectively. Close inspection of the absorption spectra of the D-A and A-D-A compounds show additional broad absorption tails in the lowest energy regime with much lower intensities. They are attributed to direct charge transfer transitions from the donor (TPA, PXZ, or DPXZ) to QX/DFQX, evidencing the presence of appreciable through-space electronic interactions.

For assignment of the emitting states, the photoluminescence spectra of the four precursors were firstly examined to identify the excited state energy levels of fragments. As illustrated in Figures 3(b) and S7, vibronically structured emissions with peak maxima (*λ*_max_) at 428−442 nm are observed for *CQX*, *CDFQX*, *C2QX*, and *C2DFQX* in nonpolar hexane, which are assigned to singlet LE states of the QX and DFQX moieties (termed ^1^LE_A_ where the subscript A denotes acceptor). The ^1^TBCT states arising from carbazole→QX/DFQX transitions are proposed to lie at higher energy levels in hexane. Increasing solvent polarity switches on their ^1^TBCT emissions in toluene (*λ*_max_ = 464–490 nm) and dichloromethane (*λ*_max_ = 556–586 nm). Taking *DPXZ-QX* as an example, new broad lower-energy emission bands appear at *λ*_max_ = 571 and 614 nm in hexane and toluene (Figure 3(c)), respectively, in comparison with the emissions of its precursor *CQX*. These bands are assigned as ^1^TSCT (DPXZ→QX) emissions. The coexistence of ^1^LE_A_/^1^TSCT and ^1^TBCT/^1^TSCT emissions in hexane and toluene reveals incomplete internal conversion (IC), presumably due to weak coupling between the TSCT and TBCT/LE states [60, 61]. It is noted that simultaneous ^1^LE_A_ and ^1^TBCT emissions have not been observed for *CQX* or *DPXZ-QX* in any solvent, revealing stronger coupling between these two states. In DCM, however, only a single emission band at *λ*_max_ = 541 nm is observed for *DPXZ-QX*. Given that the TSCT state in DCM should be further stabilized, the single band is assigned to the ^1^TBCT emission, meaning that the ^1^TSCT state in DCM is dark, which can be accounted for by a fast nonradiative decay of the TSCT state. The emission behaviours of *TPA-QX*, *PXZ-QX*, and *DPXZ-DFQX* in different solvents are akin to those of *DPXZ-QX* (Figure S8). On the basis of these findings, a diagram showing the solvent polarity-dependent emitting state(s) and their interplays is proposed in Figure 3(e) (left panel). In stark contrast, the single emission for A-D-A-type *DPXZ-2QX* and *DPXZ-2DFQX* in each solvent suggests stronger electronic communications between TSCT and TBCT/LE states which should be owing to enhanced interactions. As a result, a different excited state evolution dynamics is proposed, as shown in Figure 3(e) (right panel). Of note, the oxygen effects on the emissions of all the D-A and A-D-A emitters in fluid solutions were also preliminarily examined. As depicted in Figure S9, an enhancement of TSCT emission in the absence of air was observed in hexane, suggestive of the TADF characteristic.

**Figure 4:**
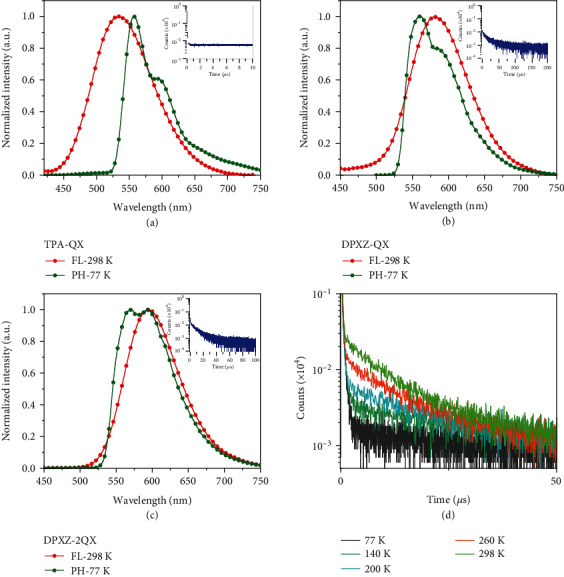
Photophysical properties in solid state. (a–c) Photoluminescence spectra and transient characteristic of (a) *TPA-QX*, (b) *DPXZ-QX*, and (c) *DPXZ-2QX* in doped mCP films (5 wt%). (d) Variable temperature transient PL decay characteristics of DPXZ-2QX in mCP films (5 wt%) at 77–298 K.

The solid-state emission properties of all the D-A and A-D-A compounds were studied in a panel of hosts including polymethyl methacrylate (PMMA), 1,3-bis(N-carbazolyl) benzene (mCP), 9,9′-biphenyl-3,3′-diylbis-9H-carbazole (mCBP), 4,4′,4^″^-tris(carbazole-9-yl) triphenylamine (TCTA), and 2,2,2^″^-(1,3,5-benzinetriyl)-tris(1phenyl-1-H-benzimidazole (TPBI). All emitters in doped films display single emissions at room temperature (Figure S10). By comparing with the emissions of their precursors (Figure S11), the emissive states for all the compounds in the solid state are assigned as TSCT in nature. Enhanced through-space electronic coupling interactions are surmised to boost the IC processes, leading to the populations of lowest-lying TSCT states. Impressively, the PLQYs for the emitters containing DPXZ are determined to be 71−91% and 65−86% in mCP and mCBP under Ar, respectively (Tables 1 and S4). In contrast, *TPA-QX* and *PXZ-QX* show much lower PLQYs (Table 1). To study their emission mechanisms, phosphorescence spectra of all emitters in different hosts at 77 K were also recorded. As depicted in Figures 4 and S10, *TPA-QX* and *PXZ-QX* have similar phosphorescence energies which are assigned to ^3^LE_A_-dominated states. Differently, the emission envelopes become less structured for *DPXZ-QX*/*DPXZ-DFQX* and *DPXZ-2QX*/*DPXZ-2DFQX*. This trend suggests an increasing ^3^CT character of the T_1_ state. As a result, the S_1_ and T_1_ states become close for these four molecules with Δ*E*_ST_ values smaller than 0.1 eV (Tables 1 and S4). In contrast, the Δ*E*_ST_ values of *TPA-QX* and *PXZ-QX* are estimated to be up to 0.40 eV. In line with the theoretical prediction, transient PL measurements of all emitters in mCP show long-lived components for those containing DPXZ, corroborating their TADF nature (Figures 4 and S12). Variable temperature transient PL characteristic of *DPXZ-2QX* clearly confirms the TADF mechanism (Figure 4(d)). To the best of our knowledge, all compounds except *TPA-QX* represent the rare examples of orange-red to red TSCT-TADF emitters in the literature [25, 28]. Remarkably, the average delayed fluorescence lifetimes (*τ*_d_) are reduced from 26.9 and 6.8 *μ*s for *DPXZ-QX* and *DPXZ-DFQX* to 8.7 and 4.9 *μ*s for *DPXZ-2QX* and *DPXZ-2DFQX*, respectively. Kinetic analysis of the excited state processes shows higher RISC rates of 4.64–8.21 × 10^−5^ s^−1^ for the A-D-A emitters in comparison with the D-A congeners (Tables 1 and S5). The presence of two close-lying ^3^CT states likely opens multiple RISC channels for *DPXZ-2QX* and *DPXZ-2DFQX* [52–56]. Notably, the delayed fluorescence lifetime is much shorter for *DPXZ-DFQX* than for *DPXZ-QX*. It has been established that an intervention of the ^3^LE state between the ^1^CT and ^3^CT states can boost the RISC rate significantly [52–56]. A slight increase of the acceptor strength in *DPXZ-DFQX* engenders more stabilized ^1,3^CT states between which the ^3^LE regulation is more pronounced. This difference is minimized for *DPXZ-2QX* and *DPXZ-2DFQX* because of the presence of another CT state which can also boost the RISC.

### 2.3. Electrochemistry

The electrochemical properties of all compounds were examined by cyclic voltammetry in DCM (Table 1). As shown in Figure S13, two quasireversible oxidations were observed for each compound with half-potentials (*E*_1/2_) in the range of 0.77−0.92 and 1.32−1.44 V versus Ag/AgCl, respectively. The first couple is assigned to the oxidation of TPA, PXZ, or DPXZ. The second couple is the carbazole-centered redox process. Irreversible waves with onset potentials (*E*_onset_) ranging from -1.41 to -1.31 V (versus Ag/AgCl) were noted in the cathodic scan, corresponding to the reductions of the QX and DFQX moieties. By referring to the redox potential of Cp_2_Fe^+/0^, the HOMO levels (ca. −5.1 eV) are estimated to be comparable for all molecules except *TPA-QX* (-5.25 eV). The deeper HOMO level for *TPA-QX* is consistent with the theoretical simulation and spectroscopic results. As expected, the presence of F atoms on the acceptor stabilizes the LUMOs for *DPXZ-DFQX* and *DPXZ-2DFQX* in comparison with *DPXZ-QX* and *DPXZ-2QX*. It is notable that *DPXZ-QX* has a slightly higher LUMO level than *TPA-QX* and *PXZ-QX*, likely due to stronger interactions between more planar DPXZ and QX.

### 2.4. Electroluminescence Performance

**Figure 5:**
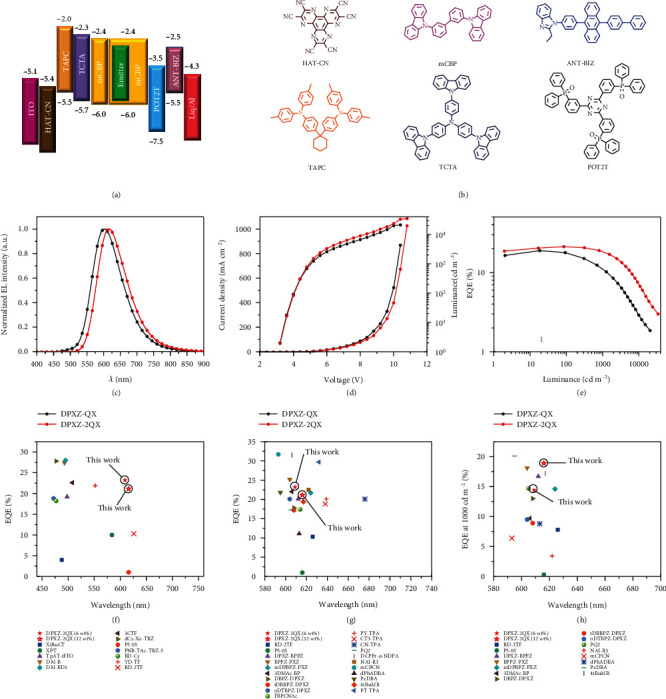
Device structure and device characteristics. (a) Device structure of the OLEDs doped with *DPXZ-QX*, *DPXZ-DFQX*, *DPXZ-2QX*, and *DPXZ-2DFQX*. (b) Chemical structures of the organic compounds used in the device fabrications. (c) Electroluminescence spectra of devices doped with *DPXZ-QX* (12 wt%) and *DPXZ-2QX* (12 wt%) at 1000 cd m^−2^. (d) J-V-L characteristics of the devices doped with *DPXZ-QX* (12 wt%) and *DPXZ-2QX* (12 wt%). (e) The plot of EQE versus luminance of the devices doped with *DPXZ-QX* (12 wt%) and *DPXZ-2QX* (12 wt%). (f) Summary of max EQE versus wavelength of representative full-colour TSCT-TADF OLEDs. (g) Max EQE and (h) EQE at 1000 cd m^−2^ versus wavelength (590−680 nm) of representative TSCT- and TBCT-TADF OLEDs.

Light-emitting devices using strongly luminescent *DPXZ-QX*, *DPXZ-DFQX*, *DPXZ-2QX*, and *DPXZ-2DFQX* as the dopants were fabricated through vacuum deposition with an architecture of ITO/HAT-CN (5 nm)/TAPC (30 nm)/TCTA (15 nm)/mCBP (10 nm)/mCBP:emitter (15 nm)/POT2T (20 nm)/ANT-BIZ (30 nm)/Liq (2 nm)/Al (100 nm) (Figure 5(a)). Chemical structures of 1,4,5,8,9,11-hexaazatriphenylene hexacarbonitrile (HAT-CN), di-[4-(*N*,*N*-ditolyl-amino)-phenyl]cyclohexane (TAPC), 4,4′,4^″^-tris(carbazole-9-yl)triphenylamine (TCTA), 9,9′-biphenyl-3,3′-diylbis-9H-carbazole (mCBP), (1,3,5-triazine-2,4,6-triyl)tris(benzene-3,1-diyl)tris(diphenylphosphine oxide) (POT2T), and 1-[4-(10-[1,1′-biphenyl]-4-yl-9-anthracenyl)phenyl]-2-ethyl-1*H*-benzimidazole (ANT-BIZ) are depicted in Figure 5(b). The neat films of HAT-CN, TAPC, TCTA, POT2T, and ANT-BIZ act as hole-injection, hole-transporting, electron/exciton blocking, hole/exciton blocking, and electron-transporting layers, respectively. An additional layer of mCBP was also inserted adjacent to the emitting layer to confine excitons. Device characteristics are plotted in Figures S14–S17 and the representative data of *DPXZ-QX* and *DPXZ-2QX* in Figure 5. The key numerical device data are compiled in Table 2 (*DPXZ-QX* and *DPXZ-2QX*) and Table S6 (*DPXZ-DFQX* and *DPXZ-2DFQX*).

The electroluminescence (EL) maxima lie at 594−599 nm, 602−617 nm, 605−616 nm, and 616−625 nm for *DPXZ-QX*, *DPXZ-DFQX*, *DPXZ-2QX*, and *DPXZ-2DFQX*, respectively, dependent on dopant concentrations. In line with their PL difference, the EL spectra of devices based on *DPXZ-2QX* and *DPXZ-2DFQX* are redshifted from their double-decker congeners. The maximum EQE/current efficiency/power efficiency are recorded as 23.2%/38.6 cd A^−1^/30.3 lm W^−1^, respectively, for the device with 6 wt% *DPXZ-2QX*. The efficiencies are twofolds higher than the previous record value for red TSCT-TADF OLEDs (Figure 5(f)) [28]. It is worth mentioning that most of the red TBCT-TADF emitters in the literature use triphenylamine (TPA) and its substituted derivatives as the donor [42–49, 62, 63]. As summarized in Table S7, the maximum EQE of 23.2% for *DPXZ-2QX* represents one of the highest efficiencies for red TADF emitters without using TPA and its substituted derivatives as the donor [43, 46, 47, 59, 64–67]. It is noted *DPXZ-DFQX* and *DPXZ-2DFQX* have lower EQEs than the emitters without containing F atoms, presumably due to the self-quenching effect for the former. The higher concentration-sensitivity of device EQEs for *DPXZ-DFQX* and *DPXZ-2DFQX* also supports this proposition. Among the four examined emitters, *DPXZ-QX* shows the largest efficiency roll-off at high luminance, which should be mainly due to its much longer delayed fluorescence lifetime. Akin to a previous finding [40], a higher concentration was found to be beneficial for reducing efficiency roll-off at high brightness. Improved charge balance is proposed to be responsible for this characteristic. With a weak concentration-quenching effect and a short delayed fluorescence lifetime, the EQE of the device doped with 12 wt% *DPXZ-2QX* remains as high as 18.9% at the luminance of 1000 cd m^−2^. It is remarkable that this performance is superior than most of the red TADF emitters, irrespective of a TBCT or TSCT process (Figure 5(h) and Table S7). For instance, despite a high maximum EQE over 30%, the value dramatically drops to 6.4% at the brightness of 1000 cd m^−2^ [68]. Therefore, the accomplishments herein demonstrate that a TSCT design integrating high molecular rigidity, strong intramolecular *π*-*π* interactions, and multiple donors/acceptors is viable to deliver high-performance red TADF devices.

## 3. Discussion

In summary, a molecular design of high-performance orange-red to red TADF emitters featuring intramolecular TSCT excited states has been demonstrated. The confining of rigid and (quasi)planar donor and acceptor(s) in a face-to-face orientation, which allows for strong intramolecular *π*-stacking interactions within the donor-acceptor pair, ensures concurrently boosted radiative charge transfer transition and suppressed nonradiative decay. Together with the regulation of the RISC rate by using multiple acceptors, an acceptor-donor-acceptor-type emitter with a sandwich configuration has been developed to deliver high-performance red OLEDs with a high external quantum efficiency and a small efficiency roll-off. This achievement substantiates the significance of control over the conformation and orientation of donor-acceptor for the design of high-efficiency TSCT-TADF emitters. The engineering of intramolecular cofacial *π*-stacking interactions provides a modular approach to the development of full-colour high-performance TADF emitters.

## Figures and Tables

**Table 1:** Photophysical and electrochemical data.

Compounds	*λ*_em_^a^ (nm)	*τ*_p_^a^ (ns)	*τ*_d_^a^ (*μ*s)	*Φ*_PL_^b^ (%)	Δ*E*_ST_^c^ (eV)	*k*_RISC_^d^ (10^5^ s^−1^)	*E*_1/2_ (ox) (V)	*E*_onset_ (red) (V)	*E*_HOMO_^e^ (eV)	*E*_LUMO_^f^ (eV)
*TPA-QX*	535	30.2	—	44	0.38	—	0.92, 1.34	-1.33	-5.25	-3.00
*PXZ-QX*	573	62.3	—	32	0.24	—	0.78, 1.32	-1.32	-5.11	-3.01
*DPXZ-QX*	582	91.0	26.9	74	0.09	1.86	0.77, 1.44	-1.38	-5.10	-2.95
*DPXZ-DFQX*	595	144.1	6.8	71	0.01	4.33	0.78, 1.43	-1.31	-5.11	-3.02
*DPXZ-2QX*	594	151.8	8.7	87	0.02	8.21	0.76, 1.41	-1.41	-5.09	-2.92
*DPXZ-2DFQX*	599	155.9	4.9	91	-0.05	4.64	0.80, 1.42	-1.31	-5.13	-3.02

^a^Fluorescence emission peak, lifetimes of prompt (*τ*_p_) and delayed (*τ*_d_) fluorescence for 5 wt% mCP films at room temperature under an argon atmosphere. ^b^Absolute PLQYs of the 5 wt% mCP films determined using an integrating sphere at room temperature under an argon atmosphere. ^c^Δ*E*_ST_ calculated from the high-energy onsets of the fluorescence spectra (room temperature) and phosphorescence spectra (77 K) of the 5 wt% mCP films. ^d^RISC rate. ^e^Estimated by *E*_HOMO_ = −*e*[*E*_1/2_(ox) − *E*_1/2_ (Fc^+^/Fc)] − 4.8 eV. ^f^Estimated by *E*_LUMO_ = −*e*[*E*_onset_(red) − *E*_1/2_ (Fc^+^/Fc)] − 4.8 eV; *E*_1/2_ (Fc^+^/Fc) = 0.47 V.

**Table 2:** Summary of key device data based on *DPXZ-QX* and *DPXZ-2QX*.

Conc.	*L* (cd m^−2^)^a^	CE (cd A^−1^)^b^	PE (lm W^−1^)^b^	EQE (%)^b^	*λ*_max_ (nm)^c^	CIE (*x*, *y*)^c^
*DPXZ-QX*						
3 wt%	11700	48.02; 9.98	44.44; 5.23	19.40; 4.52	594	0.48; 0.43
6 wt%	15400	48.60; 11.74	42.41; 6.15	20.56; 5.43	597	0.52; 0.44
12 wt%	21400	37.56; 19.91	32.78; 12.03	18.86; 10.19	599	0.56; 0.43
*DPXZ-2QX*						
3 wt%	21300	37.75; 16.60	29.65; 8.15	19.95; 9.03	605	0.56; 0.42
6 wt%	28400	38.59; 24.20	30.31; 13.58	23.16; 14.39	609	0.59; 0.41
12 wt%	35200	30.88; 27.96	24.26; 18.30	21.14; 18.91	616	0.60; 0.39

^a^Maximum luminance; ^b^values of current efficiency (CE), power efficiency (PE), and external quantum efficiency (EQE) at maximum and 1000 cd m^−2^; ^c^*λ*_max_ and CIE coordinates at 1000 cd m^−2^.

## Data Availability

All data supporting the findings of this study are presented in the article and supplementary materials. Additional data are available from the corresponding author upon reasonable request.

## Supplementary Materials

Supplementary Materials Experiments including general materials and instrumentations. Methods of single crystal analysis, theoretical simulations, device fabrication, and measurement. Syntheses and characterizations of new compounds. Figure S1: illustration of TSCT molecule design and list of selected TSCT molecules in the literature report. Figure S2: thermal properties. Tables S1-S3: crystal data. Figure S3: crystal structures. Figure S4: noncovalent interaction analysis using RDG. Figure S5: analysis of electronic structures using DFT and TDDFT calculations. Figures S6-S12 and Tables S4-S5: photophysical properties. Figure S13: cyclic voltammograms. Figures S14-S17 and Table S6: OLED data. Table S7: comparison of the present device performances with those in the literature reports.
