# Supplementary material for: Acceptor-Donor-Acceptor π-Stacking Boosts Intramolecular Through-Space Charge Transfer towards Efficient Red TADF and High-Performance OLEDs
Source: Research (Wash D C). 2022 Jun 24;2022:9892802. doi: 10.34133/2022/9892802 (PMC9275096; doi:10.34133/2022/9892802)

Supplementary Materials

Acceptor-Donor-Acceptor π-Stacking Boosts Intramolecular Through-Space Charge Transfer towards Efficient Red TADF and High Performance OLEDs

Chenglin Jiang, Jingsheng Miao, Danwen Zhang, Zhenhua Wen, Chuluo Yang, and Kai Li*

Dr. C. Jiang, Dr. J. Miao, D. Zhang, Z. Wen, Dr. Prof. C. Yang, Dr. K. Li

Shenzhen Key Laboratory of New Information Display and Storage Materials, College of Materials Science and Engineering
Shenzhen University, Shenzhen 518055, China

E-mail: kaili@szu.edu.cn

Dr. C. Jiang
College of Physics and Optoelectronic Engineering

Shenzhen University, Shenzhen 518060, China

Table of Contents

[Experimental Section 4](#_Toc92806333)

[General 4](#_Toc92806334)

[Single Crystal Analysis 4](#_Toc92806335)

[Computational Methods 5](#_Toc92806336)

[Device Fabrication and Measurement 5](#_Toc92806337)

[Synthesis and Characterization 6](#_Toc92806338)

[References 26](#_Toc92806359)

[NMR Spectra 28](#_Toc92806360)

[**Figure S1**. (a) Simplified structural analysis based on trigonometry towards the design of TSCT molecules. (b) Structures, photo- and electroluminescence data of representative TSCT emitters in the literature reports. 11](#_Toc92806342)

[**Figure S2**. TGA traces of **PXZ-QX**, **TPA-QX**, **DPXZ-QX**, **DPXZ-DFQX**, **DPXZ-2QX** and **DPXZ-2DFQX** under Ar recorded at a heating rate of 10 ^o^C min^-1^. 11](#_Toc92806342)

[**Figure S3.** The perspective view of X-ray crystal structures of **TPA-QX**, **PXZ-QX**, **DPXZ-QX**, **DPXZ-2QX**, **DPXZ-DFQX** and **DPXZ-2DFQX**. 15](#_Toc92806343)

[**Figure S4.** The RDG analysis of **TPA-QX**, **PXZ-QX**, **DPXZ-QX**, **DPXZ-DFQX**, **DPXZ-2QX** and **DPXZ-2DFQX** based on optimized ground state geometries. 16](#_Toc92806344)

[**Figure S5.** Calculated electronic structures of (a) **PXZ-QX** and **TPA-QX**, (b) **DPXZ-QX** and **DPXZ-DFQX**, and (c) **DPXZ-2QX** and **DPXZ-2DFQX**. From left to right: HOMO and LUMO contour plots in optimized S_0_ state; NTOs of the S_1_, T_1_ and T_2_ states at optmized S_0_ structure (green: hole; purpule: particle); Energy levels of the HOMO and LUMO in S_0_ state, S_1_, T_1_ and T_2_ excited state energies at optimized S_0_ structure, and the oscillator strength for S_0_→S_1_ transition. 17](#_Toc92806344)

[**Figure S6.** UV-Vis absorption spectra of (a) **PXZ-QX** and **TPA-QX** and (b) **DPXZ-QX, DPXZ-DFQX, CQX** and **CDFQX** and (c) **DPXZ-2QX**, **DPXZ-2DFQX**, **C2QX** and **C2DFQX** in toluene (concentration: ~10^-5^ M) 18](#_Toc92806344)

[**Figure S7.** Fluorescence spectra of **CDFQX**, **C2QX** and **C2DFQX** in different solvents at 298 K. 18](#_Toc92806344)

[**Figure S8**. Fluorescence spectra of **TPA-QX**, **PXZ-QX**, **DPXZ-DFQX** and **DPXZ-2DFQX** in different solvents at 298 K. 18](#_Toc92806345)

[**Figure S9**. Fluorescence (Fluo, 298 K) spectra of **DPXZ-QX**, **DPXZ-DFQX**, **DPXZ-2QX** and **DPXZ-2DFQX** in aerated and deaerated hexane. 19](#_Toc92806346)

[**Figure S10.** Photoluminescence spectra of (a) **PXZ-QX**, (b) **TPA-QX**, (c) **DPXZ-QX**, (d) **DPXZ-DFQX**, (e) **DPXZ-2QX** and (f) **DPXZ-2DFQX** in doped films (5 wt%). 20](#_Toc92806347)

[**Figure S11.** Photoluminescence spectra of **CQX**, **CDFQX**, **C2QX** and **C2DFQX** in doped mCP films at a concentration of 5 wt%. 20](#_Toc92806348)

[**Figure S12.** Transient characteristic of (a) **PXZ-QX**, (b) **TPA-QX**, (c) **DPXZ-QX**, (d) **DPXZ-DFQX**, (e) **DPXZ-2QX** and (f) **DPXZ-2DFQX** in doped mCP films (5 wt%).. 21](#_Toc92806347)

[**Figure S13.** CV curves of **PXZ-QX**, **TPA-QX**, **DPXZ-QX**, **DPXZ-DFQX**, **DPXZ-2QX** and **DPXZ-2DFQX** in dichloromethane with an Ag/AgCl standard electrode as the reference electrode. The oxidation of ferrocene occurs at E_1/2_ = 0.47 V under the same condition. 22](#_Toc92806347)

[**Figure S14.** Normalized EL spectra, EQE-luminance characteristics, and current density-luminance-voltage characteristics of OLEDs with **DPXZ-QX**-doped mCBP as the emitting layer. 23](#_Toc92806351)

[**Figure S15.** Normalized EL spectra, EQE-luminance characteristics, and current density-luminance-voltage characteristics of OLEDs with **DPXZ-DFQX**-doped mCBP as the emitting layer. 23](#_Toc92806352)

[**Figure S16.** Normalized EL spectra, EQE-luminance characteristics, and current density-luminance-voltage characteristics of OLEDs with **DPXZ-2QX**-doped mCBP as the emitting layer. 24](#_Toc92806353)

[**Figure S17.** Normalized EL spectra, EQE-luminance characteristics, and current density-luminance-voltage characteristics of OLEDs with **DPXZ-2DFQX**-doped mCBP as the emitting layer. 24](#_Toc92806354)

[**Table S1.** Crystallographic data for **DPXZ-QX** and **DPXZ-DFQX**. 12](#_Toc92806339)

[**Table S2.** Crystallographic data for **TPA-QX** and **PXZ-QX**. 13](#_Toc92806340)

[**Table S3.** Crystallographic data for **DPXZ-2QX** and **DPXZ-2DFQX**. 14](#_Toc92806341)

[**Table S4.** Summary of key photophysical data of **TPA-QX**, **PXZ-QX**, **DPXZ-QX**, **DPXZ-DFQX**, **DPXZ-2QX** and **DPXZ-2DFQX** in different hosts. 22](#_Toc92806349)

[**Tabel S5.** Summary of the excited state kinectic parameters of **DPXZ-QX**, **DPXZ-DFQX**, **DPXZ-2QX** and **DPXZ-2DFQX** in doped mCP Films. 22](#_Toc92806350)

[**Table S6.** Summary of key device data based on **DPXZ-DFQX** and **DPXZ-2DFQX**. 25](#_Toc92806355)

[**Table S7.** Device data of the doped red TADF OLEDs (EL_max_ ≥ 590 nm) based on selected representative emitters in the literature and comparison with this work. 25](#_Toc92806357)

# Experimental Section

# General

All chemicals and materials, unless otherwise noted, were commercially available and used without further purification. All solvents for reactions and photophysical measurements were of HPLC grade. The chemicals 3,6-di-*tert*-butyl-1-(4,4,5,5-tetramethyl-1,3,2-dioxaborolan-2-yl)-9H-carbazole and 3,6-di-tert-butyl-1,8-bis(4,4,5,5-tetramethyl-1,3,2-dioxaborolan-2-yl)-9H-carbazole were prepared by following literature methods.^[1,2]^

^1^H and ^13^C NMR spectra were recorded in CDCl_3_ and CD_2_Cl_2_ solvent on a 500 MHz spectrometer (Bruker Daltonics, Germany) using tetramethylsilane (TMS) as the internal standard. High-resolution mass spectrometry was performed on AB SCIEX TripleTOF 6600 (Singapore) or Agilent QTOF 6550. Elemental analyses were measured on a Vario Micro cube with CHN mode. Thermogravimetric analysis (TGA) was performed on a TGA-Q50 Instrument (TA Instruments, America) with a heating rate of 10 ^o^C/min from 60 to 800 ^o^C under an argon atmosphere.

UV-vis absorption spectra were obtained on a Shimadzu UV-2600 spectrophotometer (Shimadzu, Japan) at room temperature. Room-temperature photoluminescence spectra were measured on a Hitachi F-7100 fluorescence spectrophotometer (Hitachi, Japan). Phosphorescence spectra were measured for doped films (mCP, mCBP, PMMA, TCTA and TPBI) at 77 K. The doped film samples were prepared by spin-coating chlorobenzene solutions. The transient PL decays were measured by a single photon counting spectrometer (Picoquant, FluoTime 300, Germany). The solid state absolute photoluminescence quantum yields (PLQYs) were measured on a Hamamatsu UV-NIR absolute PL quantum yield spectrometer (C13534, Hamamatsu Photonics) equipped with a calibrated integrating sphere under argon atmosphere. Cyclic voltammetry (CV) measurements were carried out on a CHI600 electrochemical analyzer (Chenhua, China) at room temperature, with a conventional three-electrode system consisting of a glassy carbon working electrode, a platinum wire auxiliary electrode, and an Ag/AgCl standard electrode which was used as the reference electrode. Dichloromethane and tetrabutylammonium hexafluorophosphate (0.1 M) were used as the solvent and supporting electrolyte, respectively. The sweep speed was set as 100 mV s^-1^.

## Single Crystal Analysis

X-ray single-crystal diffraction data of all compounds were recorded on a Bruker D8 Venture diffractometer using Cu Kα radiation (λ = 1.54184 Å). The crystal was kept at 100.0 K during data collection. Using Olex2,^[3]^ the structure was determined with the ShelXT^[4]^ structure solution program using intrinsic phasing and refined with the ShelXL refinement package using least-squares minimization.^[5]^ Full crystallographic information in CIF format has been deposited at the Cambridge Crystallographic Data Center (CCDC) under deposition number 2109059, 2109060, 213259, 213260, 213255 and 213256.

## Computational Methods

All the density functional theory (DFT) calculations were carried out using Gaussian 09 (version D.01) package^[6]^ on a PowerLeader cluster. The ground-state calculations were fully optimized using DFT with B3LYP functional at the basis set level of 6-31G (d,p).^[7]^ The excited-state properties were obtained by time-dependent density functional theory (TD-DFT) with the basis set level of PBE0/def2-SVP at optmized S_0_ structure.^[8]^

## Device Fabrication and Measurement

Devices in this work were fabricated with a configuration of ITO/HAT-CN (5 nm)/TAPC (30 nm)/TCTA (15 nm)/mCBP (10 nm)/mCBP:emitter (15 nm)/POT2T (20 nm)/ANT-BIZ (30 nm)/Liq (2 nm)/Al (100 nm). 1,4,5,8,9,11-hexaazatriphenylene hexacarbonitrile (HAT-CN) was used as a hole-injecting layer, di-[4-(N,N-ditolyl-amino)-phenyl]cyclohexane (TAPC) was used as a hole-transporting layer, 4,4',4"-tris(carbazole-9-yl)triphenylamine (TCTA) was used as an electron/exciton blocking layer, (1,3,5-triazine-2,4,6-triyl)tris(benzene-3,1-diyl)tris(diphenylphosphine oxide) (POT2T) was used as a hole/exciton blocking layer, and 1-[4-(10-[1,1'-biphenyl]-4-yl-9-anthracenyl)phenyl]-2-ethyl-1H-benzimidazole (ANT-BIZ) acted as an electron-transporting layer. The emitting layer (EML) was constructed by using 9,9′-biphenyl-3,3′-diylbis-9H-carbazole (mCBP) the host with different doping concentrations. An additional layer of mCBP was also inserted adjacent to the emitting layer to confine excitons within the emitting layer. Before film deposition, patterned ITO substrates were cleaned with detergent, rinsed in de-ionized water, acetone, and isopropanol, and then dried in an oven for 1 h in a cleanroom. The slides were then treated in an ultraviolet-ozone chamber for 5 min. The active area of devices was 2 mm × 2 mm. In the vacuum chamber, organic materials were thermally deposited in sequence. The doping process in the emitting layer was realized by co-deposition technology. Afterwards, Liq (2 nm) and Al (100 nm) were thermally deposited. The EL spectra of devices were measured by fiber optic spectrometer (Ocean Optics USB 2000) in the normal direction. The *J-V-L* curves were investigated by a dual-channel Keithley 2614B source measure unit and a PIN-25D silicon photodiode. All the measurements were conducted at room temperature under ambient condition.

## Synthesis and Characterization


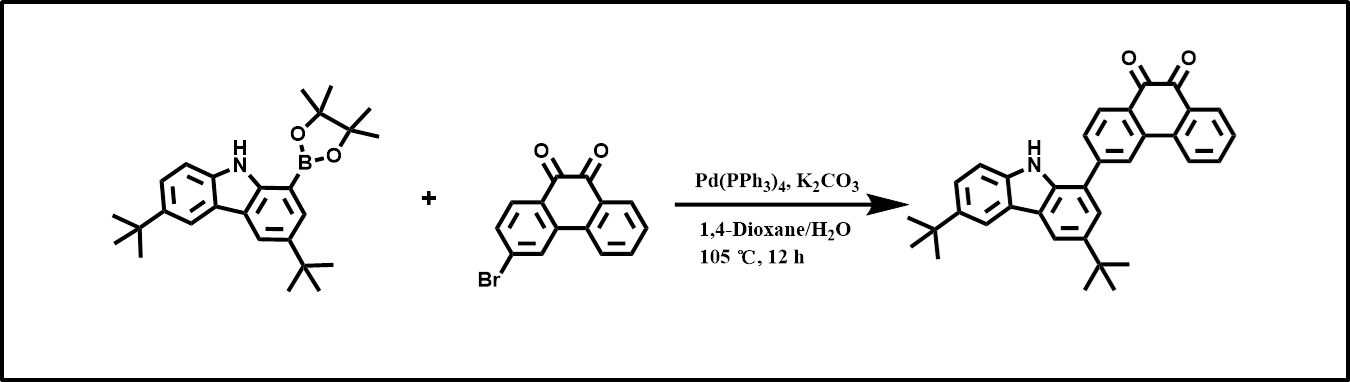


**3-(3,6-di-*tert*-butyl-9*H*-carbazol-1-yl)phenanthrene-9,10-dione**: A mixture of 3,6-di-*tert*-butyl-1-(4,4,5,5-tetramethyl-1,3,2-dioxaborolan-2-yl)-9H-carbazole (2.60 g, 6.41 mmol), 3-bromophenanthrene-9,10-dione (1.53 g, 5.33 mmol), Pd(PPh_3_)_4_ (116 mg, 0.1mmol), and K_2_CO_3_ (1.47 g, 10.66 mmol) in 1,4-dioxane/H_2_O (50 mL/50 mL) was stirred and refluxed for 12 h under argon. After cooling to room temperature, the mixture was extracted with DCM. The combined organic phase was concentrated in vacuo. The solid residue was purified by column chromatography on silica gel using DCM/petroleum ether (v/v=1/1) as the eluent to afford the product as a black powder (yield: 82%). ^1^H NMR (500 MHz, CDCl_3_) δ (ppm): 8.64 (s, 1H), 8.32 (s, 1H), 8.25 (d, *J* = 8.0 Hz, 1H), 8.20 (d, *J* = 1.5 Hz, 1H), 8.11–8.14 (m, 2H), 8.02 (d, *J* = 8.0 Hz, 1H), 7.77 (dd, *J*  = 1.0, 1.5 Hz, 1H), 7.67-7.71 (m, 1H), 7.53 (dd, *J* = 1.5, 1.5 Hz, 2H), 7.45 (m, 2H), 1.53 (s, 9H), 1.48 (s, 9H).


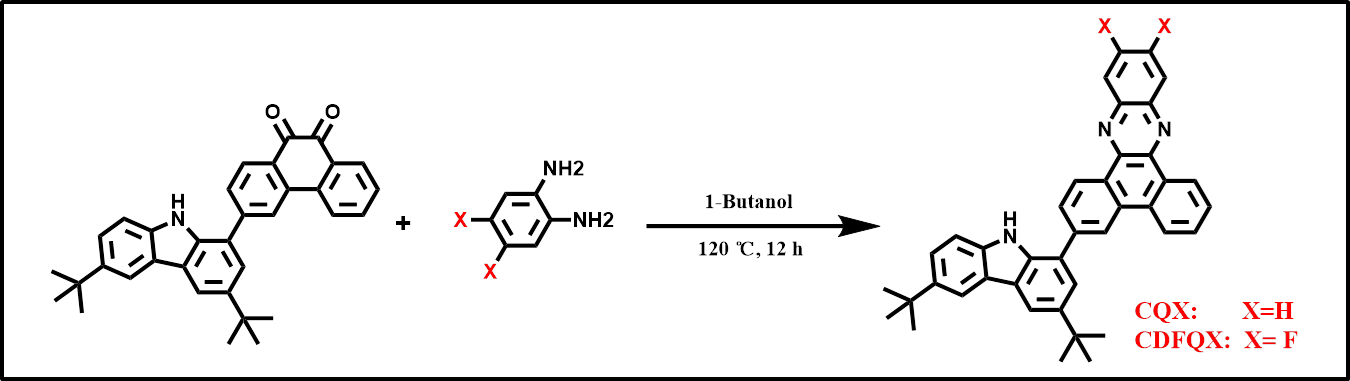


**CQX**: A mixture of 3-(3,6-di-*tert*-butyl-9*H*-carbazol-1-yl)phenanthrene-9,10-dione (1.00 g, 2.06 mmol) and benzene-1,2-diamine (267 mg, 2.47 mmol) was stirred and refluxed in 1-butanol for 12 h under argon. After reaction, the precipitate was filtered and washed with methanol, affording a yellow solid as the product (yield: 98%). ^1^H NMR (500 MHz, CDCl_3_) δ (ppm): 9.58 (d, *J* = 8.0 Hz, 1H), 9.47 (d, *J* = 9.0 Hz, 1H), 8.88 (s, 1H), 8.62 (d, *J* = 7.5 Hz, 1H), 8.37–8.40 (m, 2H), 8.28 (s, 1H), 8.17 (dd, *J* = 1.5, 1.5 Hz, 2H), 8.08 (d, *J* = 6.5 Hz, 1H), 7.88-7.91 (m, 2H), 7.78-7.84 (m, 2H), 7.67 (d, *J* = 2.0 Hz, 1H), 7.50 (dd, *J* = 2.0, 2.0 Hz, 1H), 7.37 (d, *J* = 8.5 Hz, 1H), 1.56 (s, 9H), 1.48 (s, 9H).

**CDFQX**: The procedure for synthesis of **CQX** was followed except that 4,5-difluorobenzene-1,2-diamine was used instead of benzene-1,2-diamine (yield: 90%). ^1^H NMR (500 MHz, CDCl_3_) δ (ppm): 9.48 (d, *J* = 8.0 Hz, 1H), 9.38 (dd, *J* = 1.5, 1.0 Hz, 1H), 8.86 (s, 1H), 8.61 (d, *J* = 8.0 Hz, 1H), 8.27 (s, 1H), 8.18 (dd, *J* = 1.5, 1.5 Hz, 2H), 8.05–8.10 (m, 3H), 7.77–7.84 (m, 2H), 7.67 (d, *J* = 2.0 Hz, 1H), 7.51 (dd, *J* = 2.0, 2.0 Hz, 1H), 7.37 (d, *J* = 8.5 Hz, 1H), 1.56 (s, 9H), 1.49 (s, 9H).


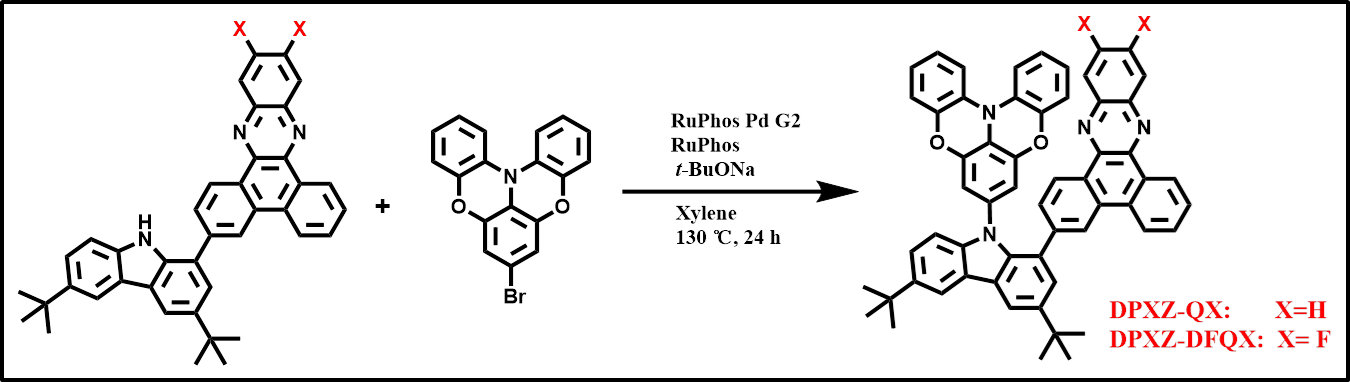


**DPXZ-QX**: A mixture of **CQX** (837 mg, 1.50 mmol), 7-bromobenzo[5,6][1,4]oxazino[2,3,4-*kl*]phenoxazine (**Br-DPXZ**, 528 mg, 1.50 mmol), RuPhos Pd G2 (116 mg, 0.15 mmol), RuPhos (70 mg, 0.15 mmol), and *t*-BuONa (288 mg, 3 mmol) in 20 mL dry xylene was stirred and refluxed for 24 h under argon. After cooling to room temperature, the mixture was filtered through a celite pad and washed with DCM. The filtrate was concentrated to dryness. The residue was purified by column chromatography on silica gel using DCM/petroleum ether (v/v=1/6) as the eluent to afford the product as a yellow powder (yield: 52%). ^1^H NMR (500 MHz, CD_2_Cl_2_) δ (ppm): 9.12 (d, *J* = 8.5 Hz, 1H), 9.05 (dd, *J* = 1.0, 1.0 Hz, 1H), 8.37 (d, *J* = 8.0 Hz, 1H), 8.29 (d, *J* = 2.0 Hz, 1H), 8.24 (s, 2H), 7.98 (dd, *J* = 8.5, 8.5 Hz, 1H), 7.92 (dd, *J* = 8.5, 8.5 Hz, 1H), 7.72-7.76 (m, 1H), 7.62-7.65 (m, 2H), 7.55 (t, *J* = 8.0, 7.0 Hz, 1H), 7.49 (dd, *J* = 2, 1.5 Hz, 1H), 7.24 (d, *J* = 9.0 Hz, 1H), 5.92-6.54 (m, 10H), 1.56 (s, 9H), 1.48 (s, 9H). ^13^C NMR (500 MHz, CD_2_Cl_2_) δ (ppm): 146.32, 145.04, 143.82, 143.41, 142.83, 142.70, 142.59, 142.10, 140.60, 136.73, 134.73, 132.14, 131.39, 131.06, 130.86, 129.99, 129.92, 129.76, 129.58, 129.01, 128.16, 126.78, 126.29, 126.07, 126.06, 125.14, 124.68, 124.65, 123.74, 123.44, 123.34, 120.00, 117.13, 116.68, 116.63, 114.47, 111.58, 110.06, 35.29, 35.24, 32.38, 32.33. HRMS (ESI) m/z: [M]^+^ calcd for C_58_H_44_N_4_O_2_, 828.3464; found 828.3464. Anal. Calcd (%) for C_58_H_44_N_4_O_2_: C, 84.03; H, 5.35; N, 6.76. Found: C, 84.16; H, 5.07; N, 6.78.

**DPXZ-DFQX**: The procedure for synthesis of **DPXZ-QX** was followed except that **CDFQX** was used instead of **CQX** (yield: 60%). ^1^H NMR (500 MHz, CD_2_Cl_2_) δ (ppm): 9.10 (d, *J* = 8.5 Hz, 1H), 9.04 (dd, *J* = 1.0, 1.0 Hz, 1H), 8.29 (d, *J* = 8.5 Hz, 1H), 8.20 (d, *J* = 2.0 Hz, 1H), 8.14-8.18 (m, 3H), 8.09-8.11 (m, 1H), 7.73-7.79 (m, 2H), 7.62-7.65 (m, 1H), 7.57 (dd, *J* = 1.5, 1.5 Hz, 1H), 7.54 (d, *J* = 2.0 Hz, 1H), 7.46 (t, 1H), 7.40 (dd, *J* = 2.0, 2.0 Hz, 1H), 7.16 (d, *J* = 8.5 Hz, 1H), 5.84-6.44 (m, 10H), 1.47 (s, 9H), 1.39 (s, 9H). ^13^C NMR (500 MHz, CD_2_Cl_2_) δ (ppm): 146.37, 146.36, 146.33, 146.31, 144.99, 143.86, 143.43, 142.82, 142.70, 142.48, 140.57, 140.07, 139.98, 139.91, 139.92, 136.68, 134.74, 132.20, 131.45, 131.18, 130.49, 129.70, 128.43, 128.27, 126.71, 126.29, 126.02, 125.93, 125.15, 124.67, 123.76, 123.40, 123.32, 119.90, 116.71, 116.69, 115.04, 114.90, 114.84, 114.70, 111.56, 110.04, 35.29, 35.25, 32.37, 32.32. HRMS (ESI) m/z: [M]^+^ calcd for C_58_H_42_F_2_N_4_O_2_, 864.3276; found 864.3328. Anal. Calcd (%) for C_58_H_42_F_2_N_4_O_2_·H_2_O: C, 78.89; H, 5.02; N, 6.35. Found: C, 78.37; H, 5.74; N, 6.25.


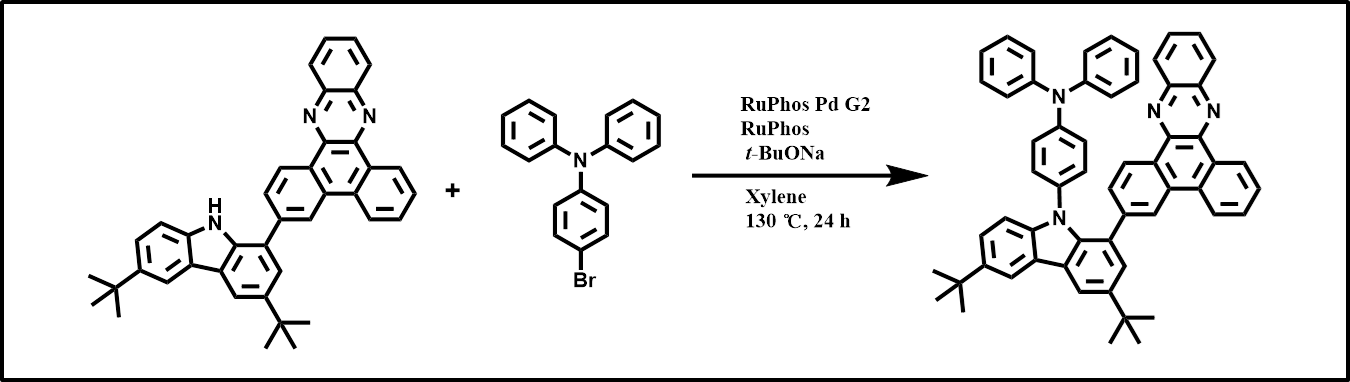


**TPA-QX**: The procedure for synthesis of **DPXZ-QX** was followed except that 4-bromo-*N,N*-diphenylaniline was used instead of **Br-DPXZ** (yield: 75%). ^1^H NMR (500 MHz, CD_2_Cl_2_) δ (ppm): 9.46 (dd, *J* = 2.0, 1.5 Hz, 1H), 9.27 (d, *J* = 3.5 Hz, 1H), 8.36-8.41 (m, 4H), 8.31 (d, *J* = 2.0 Hz, 1H), 8.26 (d, *J* = 2.0 Hz, 1H), 7.89-7.94 (m, 2H), 7.74-7.80 (m, 2H), 7.69 (dd, *J* = 1.5, 1.5 Hz, 1H), 7.60 (d, *J* = 2.0 Hz, 1H), 7.50 (dd, *J* = 2.0, 2.0 Hz, 1H), 7.27 (d, *J* = 8.5 Hz, 1H), 6.74-6.82 (m, 8H), 6.41-6.44 (m, 6H), 1.56 (s, 9H), 1.50 (s, 9H). ^13^C NMR (500 MHz, CD_2_Cl_2_) δ (ppm): 146.79, 146.01, 143.18, 142.86, 142.51, 142.35, 142.17, 142.11, 141.19, 136.69, 132.15, 131.98, 130.84, 130.41, 130.31, 129.98, 129.83, 129.45, 129.35, 128.92, 128.25, 127.94, 127.85, 126.65, 126.11, 125.37, 124.98, 124.73, 124.57, 123.94, 123.35, 123.12, 122.96, 121.20, 116.08, 109.68, 34.71, 34.66, 31.82, 31.78. HRMS (+ESI) m/z: [M+H]^+^ calcd for C_58_H_49_N_4_, 801.3952; found 801.3970. Anal. Calcd (%) for C_58_H_48_N_4_: C, 86.97; H, 6.04; N, 6.99. Found: C, 86.58; H, 6.00; N, 6.70.


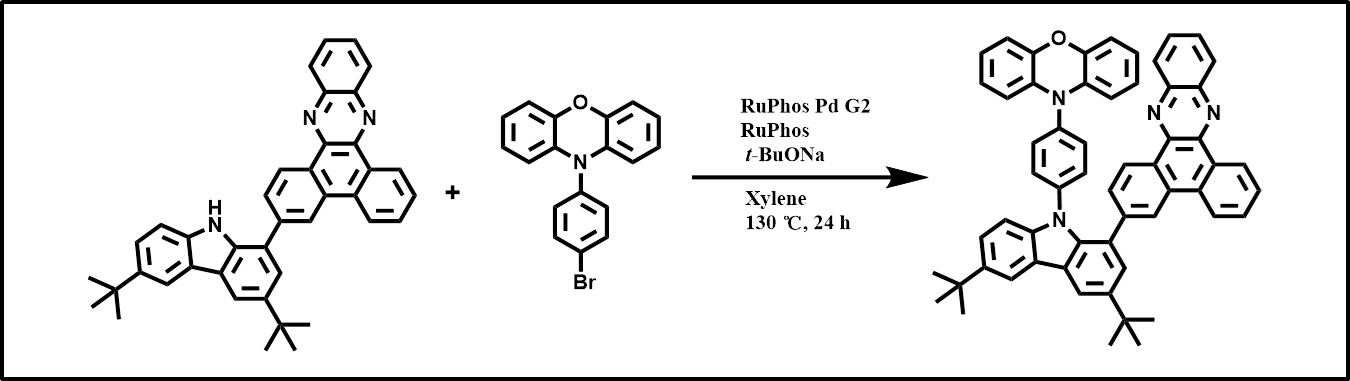


**PXZ-QX**: The procedure for synthesis of **DPXZ-QX** was followed except that 10-(4-bromophenyl)-10*H*-phenoxazine was used instead of **Br-DPXZ** (yield: 70%). ^1^H NMR (500 MHz, CD_2_Cl_2_) δ (ppm): 9.39 (dd, *J* = 1.5, 1.5 Hz, 1H), 9.28 (d, *J* = 8.5 Hz, 1H), 8.53 (d, *J* = 1.5 Hz, 1H), 8.47 (d, *J* = 7.5 Hz, 1H), 8.32-8.36 (m, 3H), 8.28 (d, *J* = 1.5 Hz, 1H), 7.89-7.92 (m, 2H), 7.74-7.81 (m, 3H), 7.62 (d, *J* = 2.0 Hz, 1H), 7.38-7.56 (m, 4H), 6.96 (d, *J* = 7.0 Hz, 2H), 6.38 (dd, *J* = 1.5, 1.5 Hz, 2H), 6.21 (td, *J* = 1.5, 1.0, 1.5 Hz, 2H), 5.80 (td, *J* = 1.5, 1.0, 1.5 Hz, 2H), 5.26 (dd, *J* = 1.5, 1.5 Hz, 2H), 1.57 (s, 9H), 1.51 (s, 9H). ^13^C NMR (500 MHz, CD_2_Cl_2_) δ (ppm): 144.04, 143.96, 143.55, 142.41, 142.22, 142.19, 142.14, 142.04, 141.25, 139.37, 136.70, 136.61, 133.76, 131.79, 131.14, 131.00, 130.40, 130.35, 130.02, 129.89, 129.40, 129.28, 128.92, 128.51, 128.08, 127.13, 126.14, 125.95, 125.73, 125.56, 124.23, 123.72, 123.07, 122.91, 120.86, 116.33, 116.27, 114.91, 112.57, 109.78, 34.79, 34.71, 31.78, 31.74. HRMS (+ESI) m/z: [M+H]^+^ calcd for C_58_H_47_N_4_O, 815.3744; found 815.3764. Anal. Calcd (%) for C_58_H_46_N_4_O·(CH_2_Cl_2_)_0.5_: C, 81.94; H, 5.52; N, 6.53. Found: C, 82.26; H, 5.11; N, 6.66.


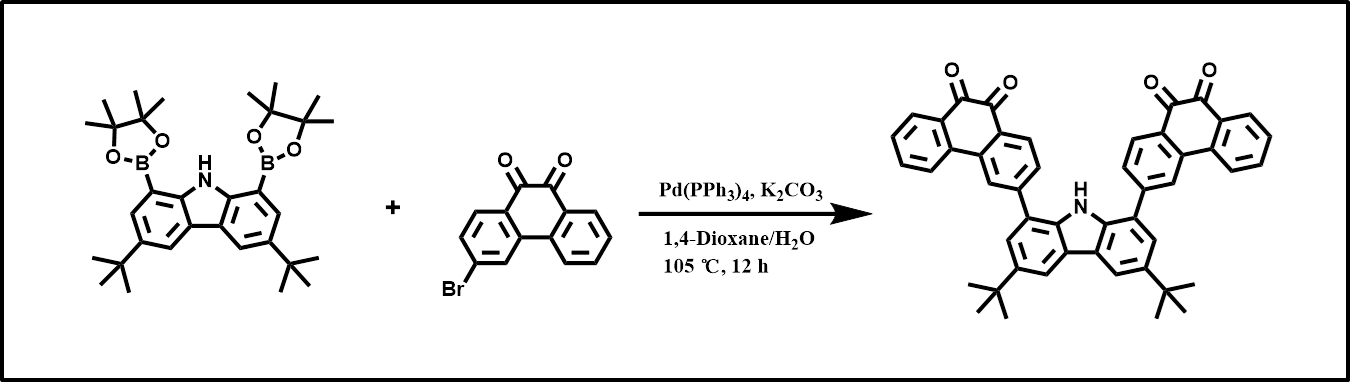


**3,3'-(3,6-di-tert-butyl-9*H*-carbazole-1,8-diyl)bis(phenanthrene-9,10-dione)**: A mixture of 3,6-di-tert-butyl-1,8-bis(4,4,5,5-tetramethyl-1,3,2-dioxaborolan-2-yl)-9H-carbazole (2.60 g, 4.89 mmol), 3-bromophenanthrene-9,10-dione (3.51 g, 12.23 mmol), Pd(PPh_3_)_4_ (116 mg, 0.1mmol), and K_2_CO_3_ (1.35 g, 9.78 mmol) in 1,4-Dioxane/H_2_O (50 mL/50 mL) was stirred and refluxed for 12 h under argon. After cooling to room temperature, the mixture was extracted with DCM. The combined organic phase was concentrated in vacuo. The solid residue was purified by column chromatography on silica gel using DCM/petroleum ether (v/v=2/1) as the eluent to afford the product as a black powder (yield: 75%). ^1^H NMR (500 MHz, CDCl_3_) δ (ppm): 10.02 (s, 1H), 8.27 (s, 2H), 8.04 (s, 2H), 7.86 (d, *J* = 6.5 Hz, 2H), 7.64 (d, *J* = 7.5 Hz, 4H), 7.46-7.51 (m, 4H), 7.35 (t, *J* = 7.5, 8.0 Hz, 2H), 7.00 (t, *J* = 7.5, 7.5 Hz, 2H), 1.56 (s, 18H).


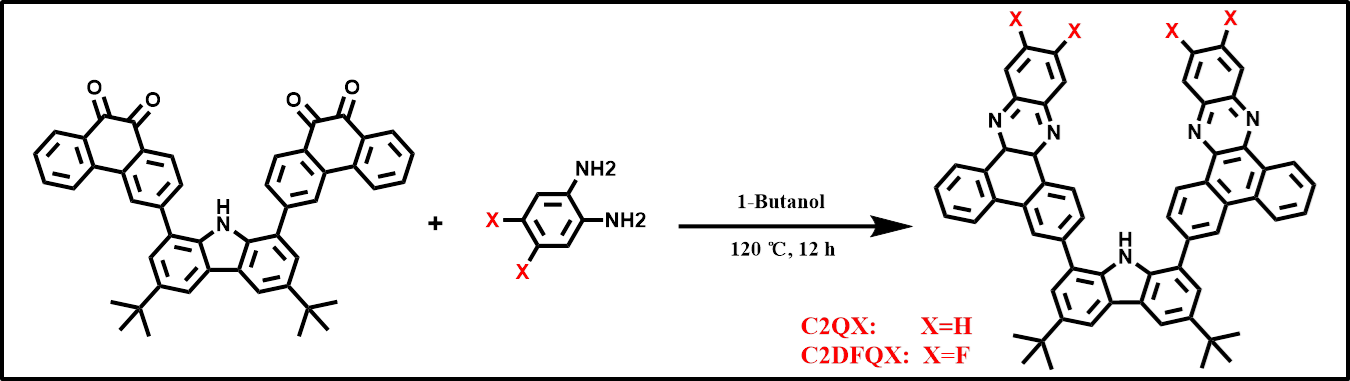


**C2QX**: A mixture of 3,3'-(3,6-di-tert-butyl-9*H*-carbazole-1,8-diyl)bis(phenanthrene-9,10-dione) (1.50 g, 2.17 mmol) and benzene-1,2-diamine (563 mg, 5.21 mmol) was stirred and refluxed in 1-butanol for 12 h under argon. After reaction, the precipitate was filtered and washed with methanol, affording a yellow solid as the product (yield: 95%). ^1^H NMR (500 MHz, CDCl_3_) δ (ppm): 9.45 (d, *J* = 10.0 Hz, 2H), 9.34 (d, *J* = 9.5 Hz, 2H), 8.86 (s, 2H), 8.65 (s, 1H), 8.56 (d, *J* = 10.5 Hz, 2H), 8.25-8.29 (m, 6H), 8.11 (d, *J* = 10.0 Hz, 2H), 7.82 (dd, *J* = 4.5, 4.5 Hz, 4H), 7.73-7.77 (m, 4H), 7.69 (t, *J* = 9.5, 9.0 Hz, 2H), 1.61 (s, 18H).

**C2DFQX**: The procedure for synthesis of **C2QX** was followed except that 4,5-difluorobenzene-1,2-diamine was used instead of benzene-1,2-diamine (yield: 82%). ^1^H NMR (500 MHz, CDCl_3_) δ (ppm): 9.21 (d, *J* = 8.0 Hz, 2H), 9.15 (d, *J* = 7.0 Hz, 2H), 8.74 (s, 2H), 8.67 (s, 1H), 8.46 (d, *J* = 8.0 Hz, 2H), 8.28 (s, 2H), 8.05 (d, *J* = 8.0 Hz, 2H), 7.89 (t, 10.5, 8.0 Hz, 2H), 7.61-7.78 (m, 8H), 1.61 (s, 18H).


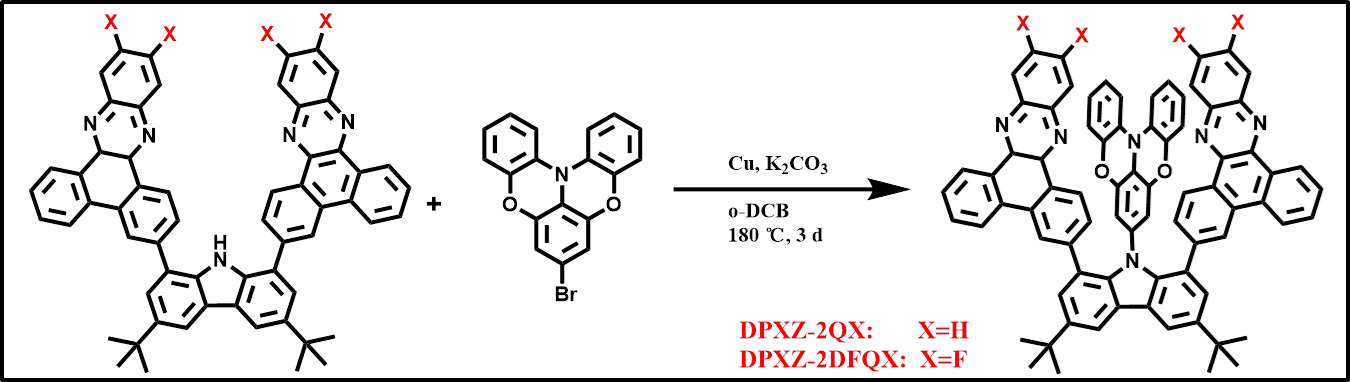


**DPXZ-2QX**: A mixture of **C2QX** (1.26 g, 1.50 mmol), 7-bromobenzo[5,6][1,4]oxazino[2,3,4-*kl*]phenoxazine (**Br-DPXZ**, 528 mg, 1.50 mmol), Cu powder (288 mg, 4.50 mmol), and K_2_CO_3_ (415 mg, 3 mmol) in 30 mL dry o-dichlorobenzene was stirred and refluxed for 72h under argon. After cooling to room temperature, the crude product was filtered through a celite pad and washed with DCM. The filtrate was concentrated to dryness. The residue was purified by column chromatography on silica gel using DCM/petroleum ether (v/v=1/4) as the eluent to afford the product as a yellow powder (yield: 61%). ^1^H NMR (500 MHz, CD_2_Cl_2_) δ (ppm): 9.00 (ddd, *J* = 8.0, 8.0, 8.0, 8.0 Hz, 4H), 8.39 (d, *J* = 2.0 Hz, 2H), 8.00-8.31 (m, 8H), 7.73-7.78 (m, 4H), 7.31-7.62 (m, 8H), 5.46-5.99 (m, 10H), 1.58 (s, 18H). The ^13^C NMR spectrum was not recorded due to its poor solubility. HRMS (+ESI) m/z: [M+H]^+^ calcd for C_78_H_55_N_6_O_2_, 1107.4381; found 1107.4396. Anal. Calcd (%) for C_78_H_54_N_6_O_2_: C, 84.61; H, 4.92; N, 7.59. Found: C, 84.73; H, 4.38; N, 7.65.

**DPXZ-2DFQX**: The procedure for synthesis of **DPXZ-2QX** was followed except that **C2DFQX** was used instead of **C2QX** (yield: 69%). ^1^H NMR (500 MHz, CDCl_2_) δ (ppm): 8.90 (ddd, *J* = 8.5, 7.5, 8.0, 8.0 Hz, 4H), 8.39 (d, *J* = 2.0 Hz, 2H), 8.26 (d, *J* = 8.0 Hz, 1H) 8.18 (s, 1H), 8.06 (d, *J* = 8.0 Hz, 1H), 7.97 (s, 1H), 7.73-7.87 (m, 4H), 7.57-7.62 (m, 4H), 7.48 (dt, *J* = 7.0, 7.5 Hz, *J* = 7.5, 7.5 Hz, 2H), 7.29-7.40 (m, 2H), 5.58-6.02 (m, 10H), 1.59 (s, 18H). The ^13^C NMR spectrum was not recorded due to its poor solubility. HRMS (+ESI) m/z: [M+H]^+^ calcd for C_78_H_51_N_6_F_4_O_2_, 1179.4004; found 1179.4001. Anal. Calcd (%) for C_78_H_50_N_6_F_4_O_2_: C, 79.44; H, 4.27; N, 7.13. Found: C, 79.43; H, 4.00; N, 7.18.


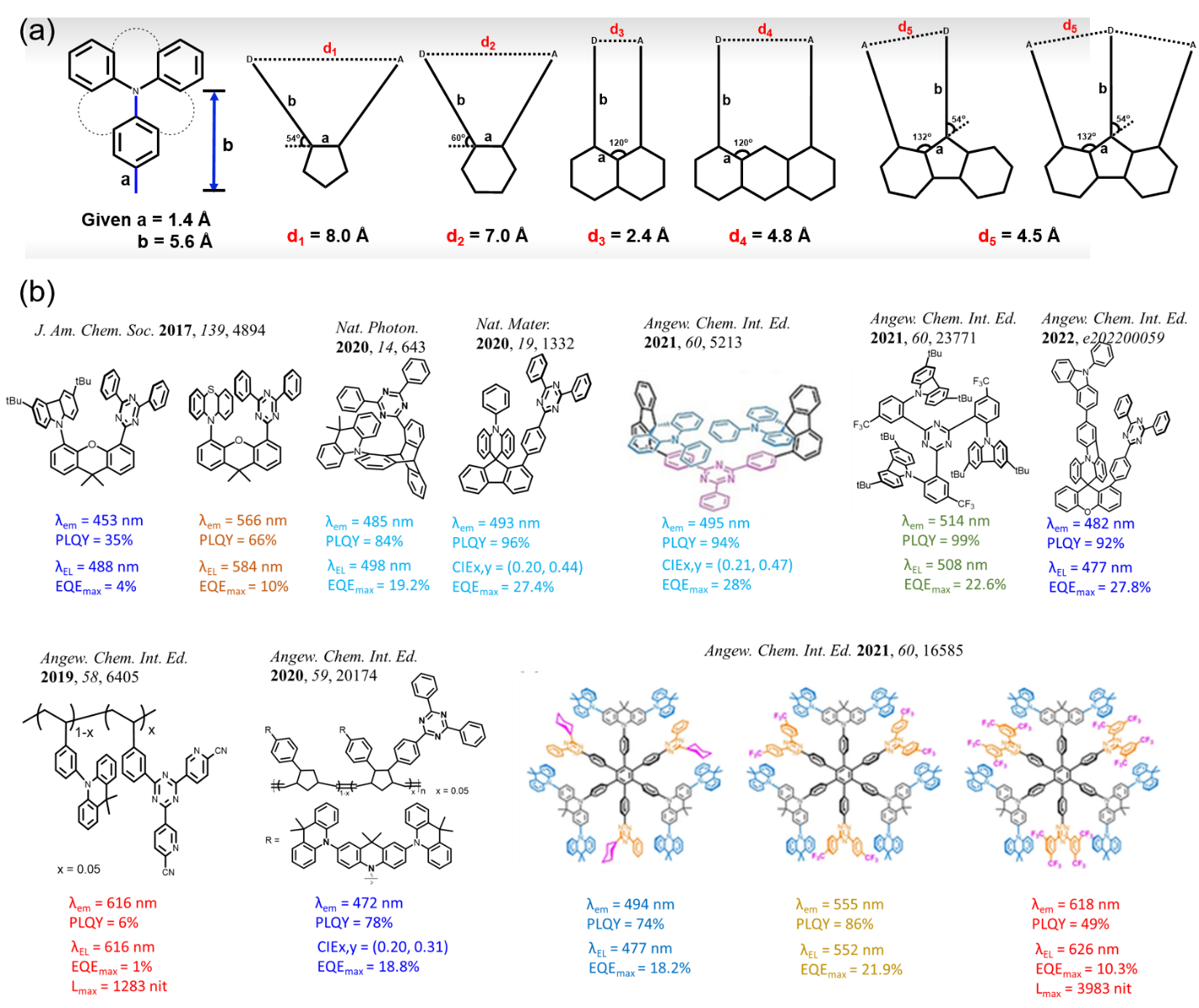


**Figure S1.** (a) Simplified structural analysis based on trigonometry towards the design of TSCT molecules. (b) Structures, photo- and electroluminescence data of representative TSCT emitters in the literature reports.


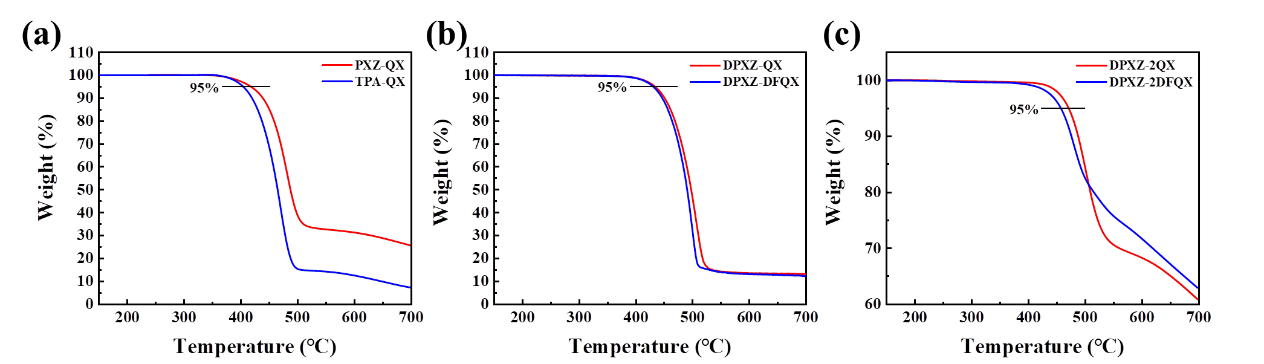


# **Figure S2.** TGA traces of **PXZ-QX**, **TPA-QX**, **DPXZ-QX**, **DPXZ-DFQX**, **DPXZ-2QX** and **DPXZ-2DFQX** under Ar recorded at a heating rate of 10 ^o^C min^-1^.

# Table S1. Crystallographic data for DPXZ-QX and DPXZ-DFQX.

| Compound | **DPXZ-QX** | **DPXZ-DFQX** |
| --- | --- | --- |
| *CCDC NO.* | 2109059 | 2109060 |
| *Formula* | C_58_H_44_N_4_O_2_ | C_58_H_42_F_2_N_4_O_2_ |
| *Formula weight* | 829.02 | 864.95 |
| *Crystal system* | orthorhombic | monoclinic |
| *Space group* | Pbca | P2_1_/c |
| *a/Å* | 17.8562(6) | 18.146(3) |
| *b/Å* | 14.0891(6) | 17.795(3) |
| *c/Å* | 36.3165(12) | 14.214(2) |
| *α/°* | 90 | 90 |
| *β/°* | 90 | 95.222(5) |
| *γ/°* | 90 | 90 |
| *Volume/Å^3^* | 9136.4(6) | 4570.7(12) |
| *Z* | 8 | 4 |
| *ρ_calc_g/cm^3^* | 1.329 | 1.257 |
| *μ/mm^‑1^* | 0.193 | 0.082 |
| *F(000)* | 3824.0 | 1808.0 |
| *Crystal size/mm^3^* | 0.16 × 0.11 × 0.08 | 0.15 × 0.12 × 0.08 |
| *2Θ range (deg)* | 3.85-52.772 | 4.174-50.752 |
| *GOF on F^2^* | 1.034 | 1.004 |
| *R_1_/wR_2_ [I>=2σ (I)]* | 0.0826/ 0.2120 | 0.0735/ 0.1645 |

# Table S2. Crystallographic data for TPA-QX and PXZ-QX.

| Compound | **TPA-QX** | **PXZ-QX** |
| --- | --- | --- |
| *CCDC NO.* | 2131259 | 2131260 |
| *Formula* | C_58_H_48_N_4_ | C_58_H_46_N_4_O |
| *Formula weight* | 801.00 | 815.03 |
| *Crystal system* | triclinic | monoclinic |
| *Space group* | P-1 | P2_1_/n |
| *a/Å* | 13.0095(13) | 10.4242(5) |
| *b/Å* | 13.3914(13) | 30.7056(17) |
| *c/Å* | 14.4412(15) | 14.2857(7) |
| *α/°* | 116.077(3) | 90 |
| *β/°* | 99.219(4) | 96.566(2) |
| *γ/°* | 99.933(4) | 90 |
| *Volume/Å^3^* | 2145.2(4) | 4542.6(4) |
| *Z* | 2 | 4 |
| *ρ_calc_g/cm^3^* | 1.240 | 1.316 |
| *μ/mm^‑1^* | 0.072 | 0.192 |
| *F(000)* | 848.0 | 1888.0 |
| *Crystal size/mm^3^* | 0.15×0.09×0.04 | 0.08×0.05×0.04 |
| *2Θ range (deg)* | 3.97 to 52.884 | 3.908 to 52.774 |
| *GOF on F^2^* | 1.052 | 1.031 |
| *R_1_/wR_2_ [I>=2σ (I)]* | 0.0531/0.1041 | 0.0975/0.2648 |

# Table S3. Crystallographic data for DPXZ-2QX and DPXZ-2DFQX.

| Compound | **DPXZ-2QX** | **DPXZ-2DFQX** |
| --- | --- | --- |
| *CCDC NO.* | 2131255 | 2131256 |
| *Formula* | C_78_H_54_N_6_O_2_ | C_78_H_50_F_4_N_6_O_2_ |
| *Formula weight* | 1107.27 | 1179.29 |
| *Crystal system* | triclinic | monoclinic |
| *Space group* | P-1 | P2_1_/n |
| *a/Å* | 12.6297(5) | 16.4384(19) |
| *b/Å* | 15.5589(6) | 14.9327(15) |
| *c/Å* | 15.7910(6) | 26.322(3) |
| *α/°* | 105.0060(10) | 90 |
| *β/°* | 104.3790(10) | 107.964(3) |
| *γ/°* | 102.9470(10) | 90 |
| *Volume/Å^3^* | 2761.98(19) | 6146.3(12) |
| *Z* | 2 | 4 |
| *ρ_calc_g/cm^3^* | 1.331 | 1.366 |
| *μ/mm^‑1^* | 0.081 | 0.175 |
| *F(000)* | 1160.0 | 2616.0 |
| *Crystal size/mm^3^* | 0.15×0.08×0.05 | 0.08×0.03×0.02 |
| *2Θ range (deg)* | 3.77 to 50.77 | 3.176 to 48.812 |
| *GOF on F^2^* | 1.038 | 0.985 |
| *R_1_/wR_2_ [I>=2σ (I)]* | 0.0558/0.1167 | 0.0691/0.1425 |

**
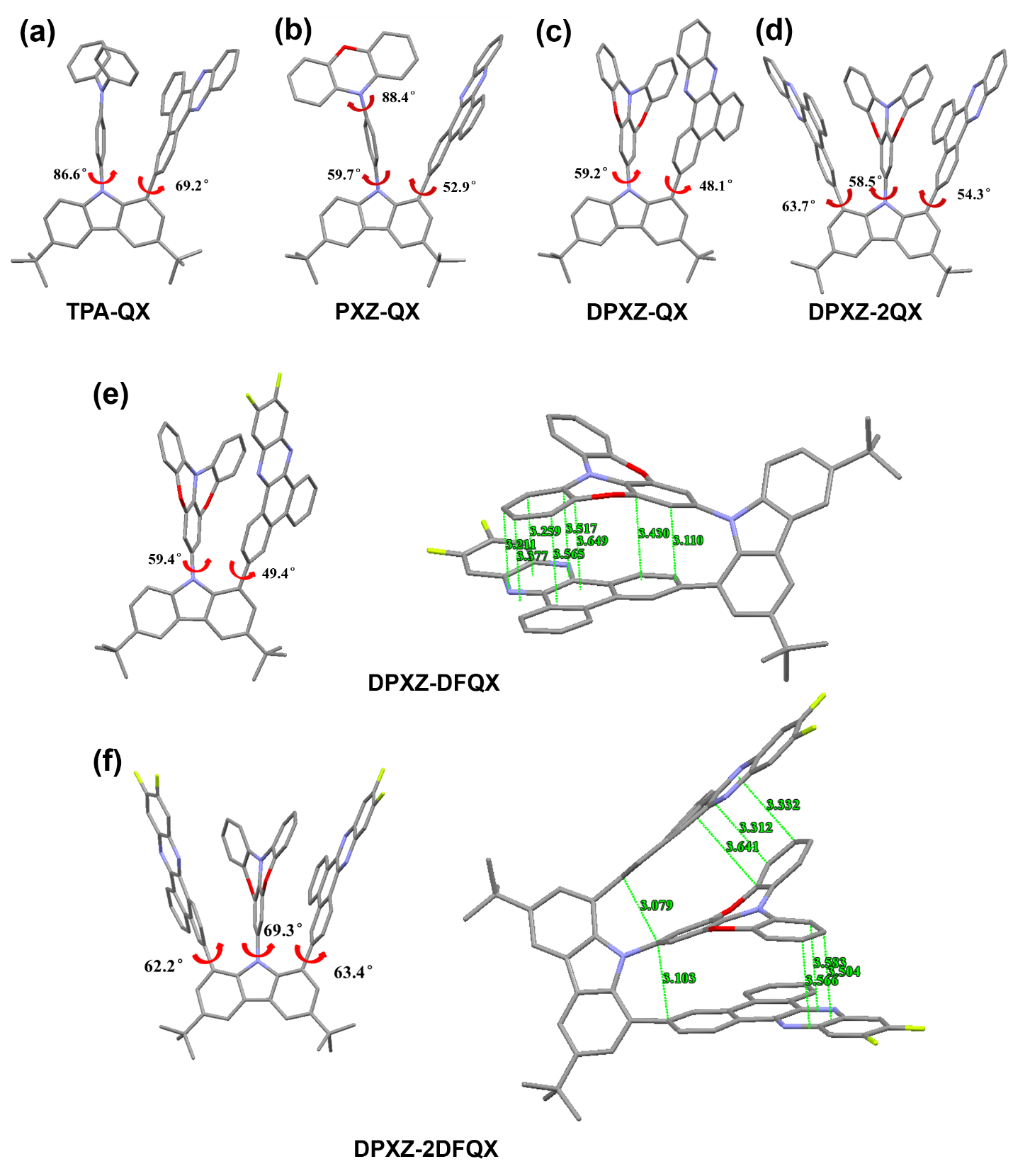
**

# Figure S3. The perspective view of X-ray crystal structures of TPA-QX, PXZ-QX, DPXZ-QX, DPXZ-2QX, DPXZ-DFQX and DPXZ-2DFQX.

#

# Figure S4. The RDG analysis of TPA-QX, PXZ-QX, DPXZ-QX, DPXZ-DFQX, DPXZ-2QX and DPXZ-2DFQX based on optimized ground state geometries.

**
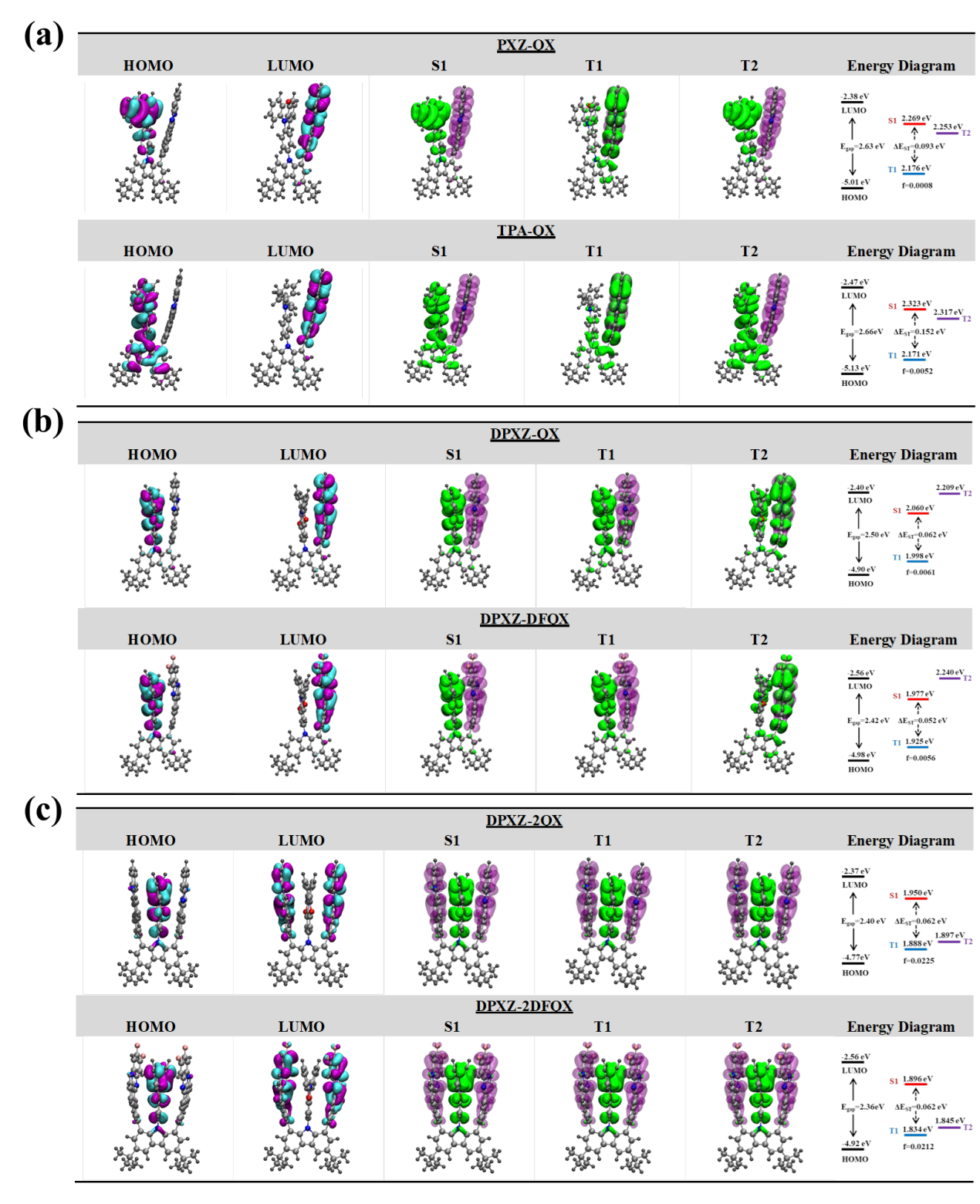
**

Figure S5. Calculated electronic structures of (a) PXZ-QX and TPA-QX, (b) DPXZ-QX and DPXZ-DFQX, and (c) DPXZ-2QX and DPXZ-2DFQX. From left to right: HOMO and LUMO contour plots in optimized S_0_ state; NTOs of the S_1_, T_1_ and T_2_ states at optimized S_0_ structure (green: hole; purple: particle); Energy levels of the HOMO and LUMO in S_0_ state, S_1_, T_1_ and T_2_ excited state energies at optimized S_0_ structure, and the oscillator strength for S_0_→S_1_ transition.

**
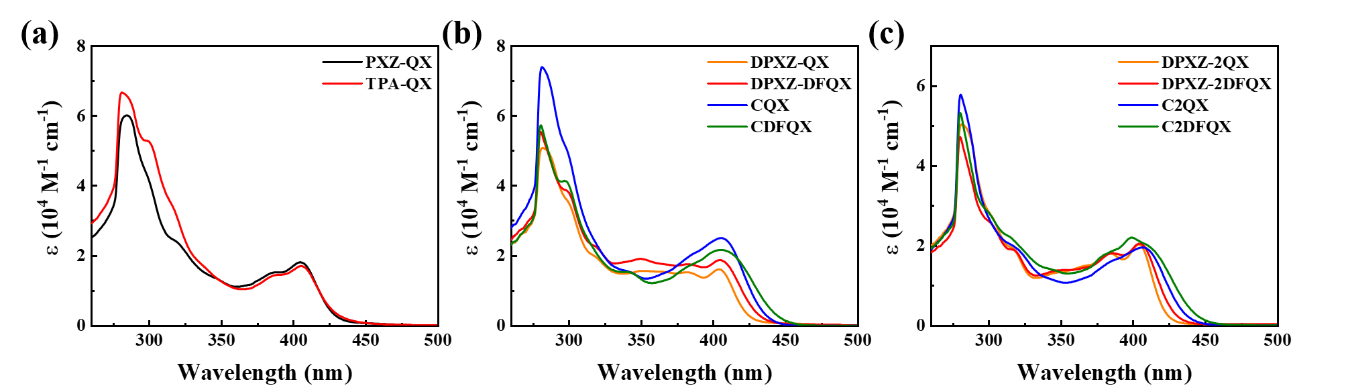
**

Figure S6. UV-Vis absorption spectra of (a) PXZ-QX and TPA-QX and (b) DPXZ-QX, DPXZ-DFQX, CQX and CDFQX and (c) DPXZ-2QX, DPXZ-2DFQX, C2QX and C2DFQX in toluene (concentration: ~10^-5^ M).


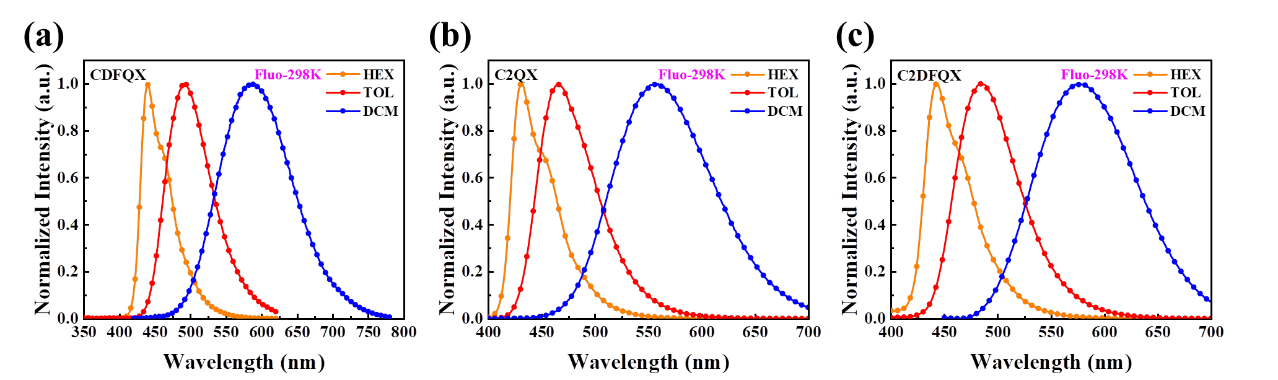


Figure S7. Fluorescence spectra of CDFQX, C2QX and C2DFQX in different solvents at 298 K.


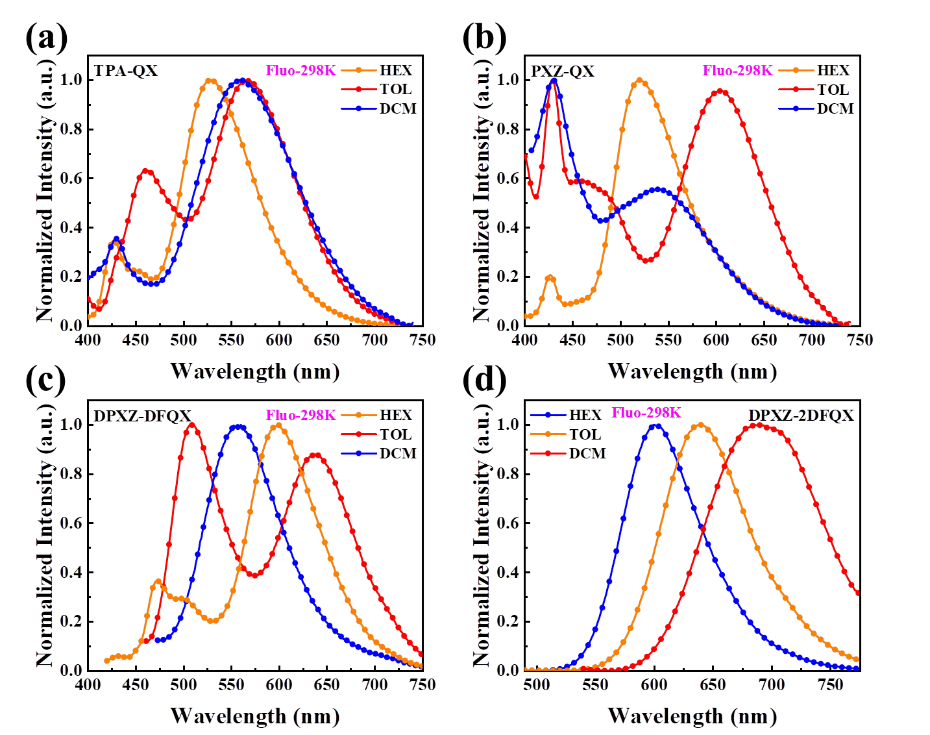


Figure S8. Fluorescence spectra of TPA-QX, PXZ-QX, DPXZ-DFQX and DPXZ-2DFQX in different solvents at 298 K.


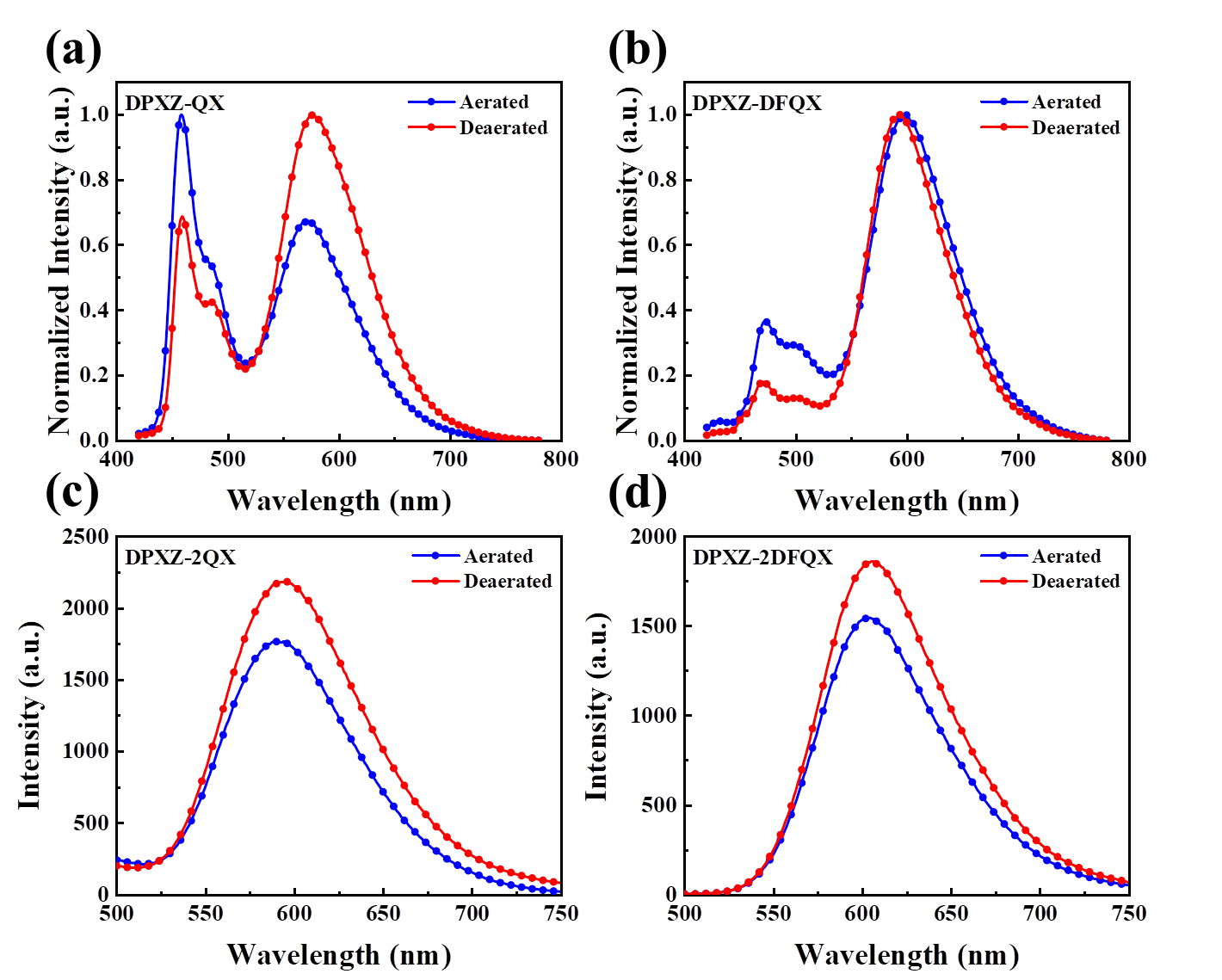


# **Figure S9.** Fluorescence (Fluo, 298 K) spectra of **DPXZ-QX**, **DPXZ-DFQX**, **DPXZ-2QX** and **DPXZ-2DFQX** in aerated and deaerated hexane.


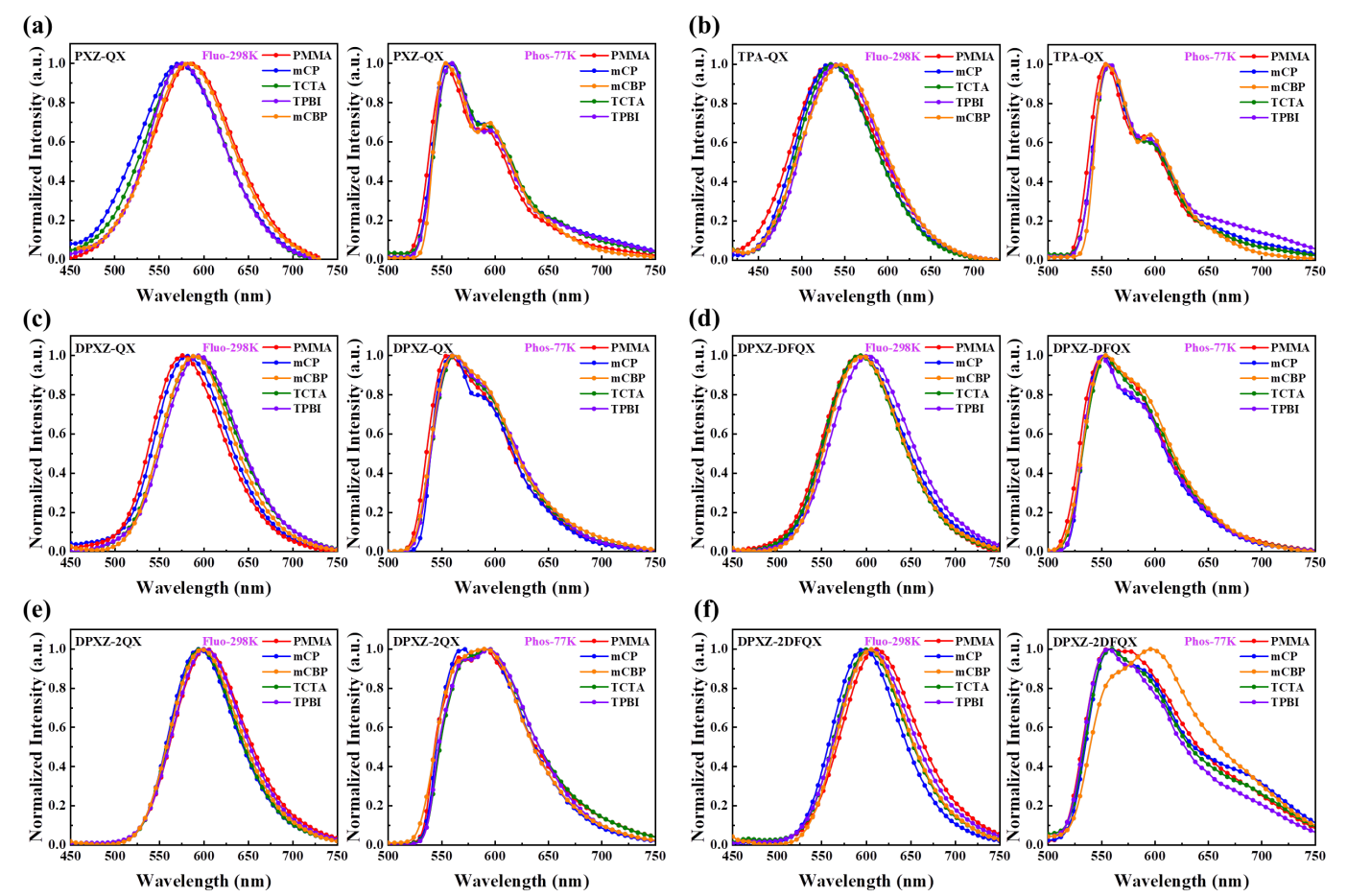


# Figure S10. Photoluminescence spectra of (a) PXZ-QX, (b) TPA-QX, (c) DPXZ-QX, (d) DPXZ-DFQX, (e) DPXZ-2QX and (f) DPXZ-2DFQX in doped films (5 wt%).

**
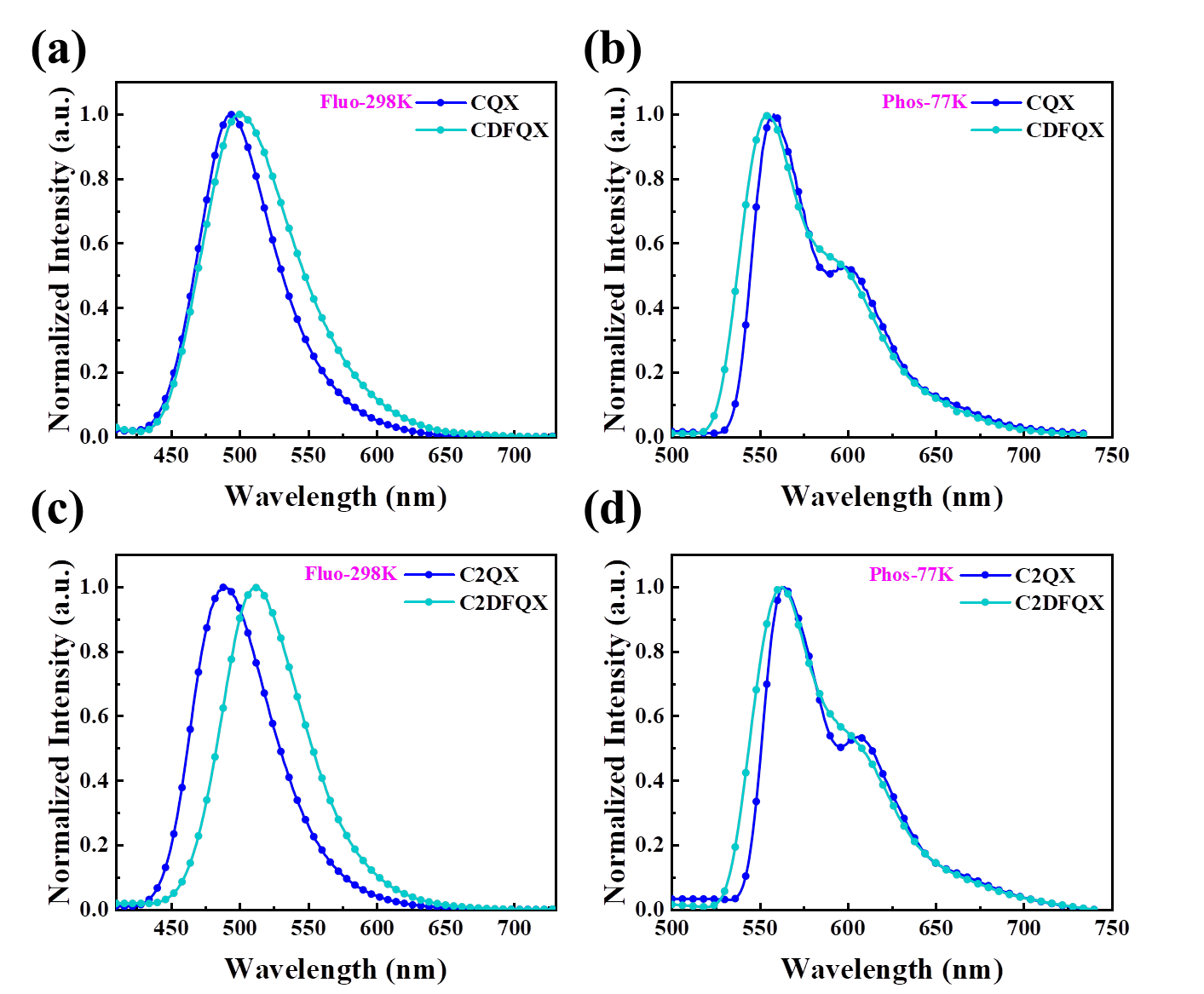
**

Figure S11. Photoluminescence spectra of CQX, CDFQX, C2QX and C2DFQX in doped mCP films at a concentration of 5 wt%.


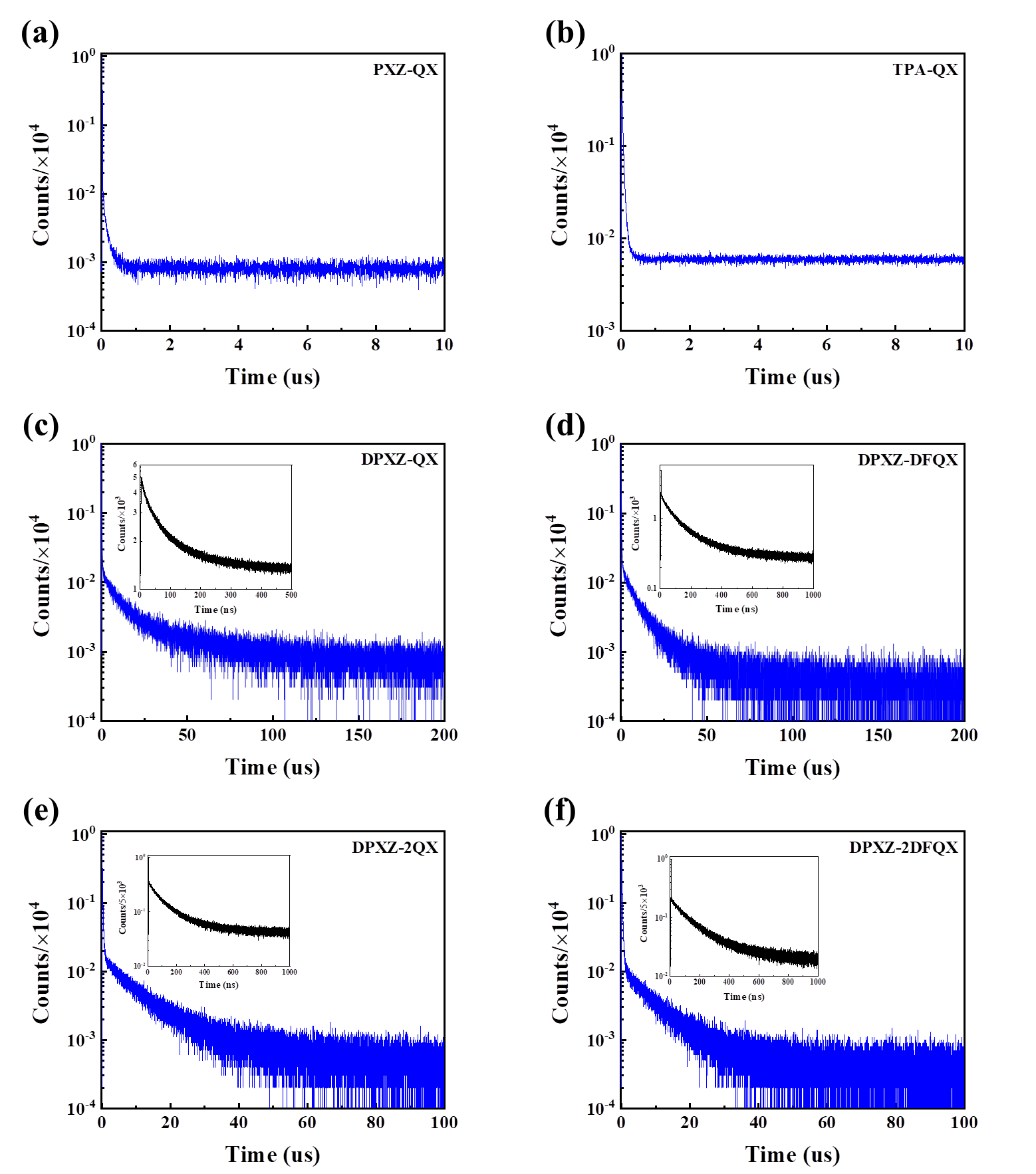


# Figure S12. Transient characteristic of (a) PXZ-QX, (b) TPA-QX, (c) DPXZ-QX, (d) DPXZ-DFQX, (e) DPXZ-2QX and (f) DPXZ-2DFQX in doped mCP films (5 wt%).

# Table S4. Summary of key photophysical data of TPA-QX, PXZ-QX, DPXZ-QX, DPXZ-DFQX, DPXZ-2QX and DPXZ-2DFQX in different hosts.

| Emitter | PMMA | | mCP | | mCBP | | | TCTA | | TPBI | |
| --- | --- | --- | --- | --- | --- | --- | --- | --- | --- | --- | --- |
|  | S_1_ | T_1_ | S_1_ | T_1_ | S_1_ | T_1_ | Φ_PL_ | S_1_ | T_1_ | S_1_ | T_1_ |
| **TPA-QX** | 2.76 | 2.36 | 2.72 | 2.34 | 2.69 | 2.32 | - | 2.67 | 2.33 | 2.66 | 2.33 |
| **PXZ-QX** | 2.55 | 2.34 | 2.58 | 2.34 | 2.54 | 2.32 | - | 2.55 | 2.34 | 2.49 | 2.33 |
| **DPXZ-QX** | 2.43 | 2.34 | 2.42 | 2.33 | 2.41 | 2.36 | 69 | 2.41 | 2.35 | 2.40 | 2.35 |
| **DPXZ-DFQX** | 2.41 | 2.40 | 2.39 | 2.38 | 2.38 | 2.37 | 65 | 2.38 | 2.37 | 2.38 | 2.37 |
| **DPXZ-2QX** | 2.34 | 2.33 | 2.34 | 2.32 | 2.33 | 2.31 | 86 | 2.33 | 2.32 | 2.33 | 2.31 |
| **DPXZ-2DFQX** | 2.32 | 2.39 | 2.33 | 2.38 | 2.31 | 2.37 | 85 | 2.33 | 2.38 | 2.33 | 2.38 |

# Table S5. Summary of the excited state kinetic parameters of DPXZ-QX, DPXZ-DFQX, DPXZ-2QX and DPXZ-2DFQX in doped mCP Films.

|  | Φ_PL_^a^  (%) | τ_p_ ^b^  (ns) | τ_d_ ^b^  (μs) | Φ_p_/Φ_d_ | *k*_r,S_  (×10^6^ s^-1^) | *k*_nr,S_  (×10^5^ s^-1^) | *k*_ISC_  (×10^6^ s^-1^) | *k*_RISC_  (×10^5^ s^-1^) |
| --- | --- | --- | --- | --- | --- | --- | --- | --- |
| **DPXZ-QX** | 74 | 91.0 | 26.9 | 0.15/0.59 | 1.65 | 5.79 | 8.79 | 1.86 |
| **DPXZ-DFQX** | 71 | 144.1 | 6.8 | 0.24/0.47 | 1.74 | 7.12 | 4.58 | 4.33 |
| **DPXZ-2QX** | 87 | 151.8 | 8.7 | 0.12/0.75 | 0.89 | 1.33 | 5.67 | 8.21 |
| **DPXZ-2DFQX** | 91 | 155.9 | 4.9 | 0.40/0.51 | 2.67 | 2.64 | 3.59 | 4.64 |

**
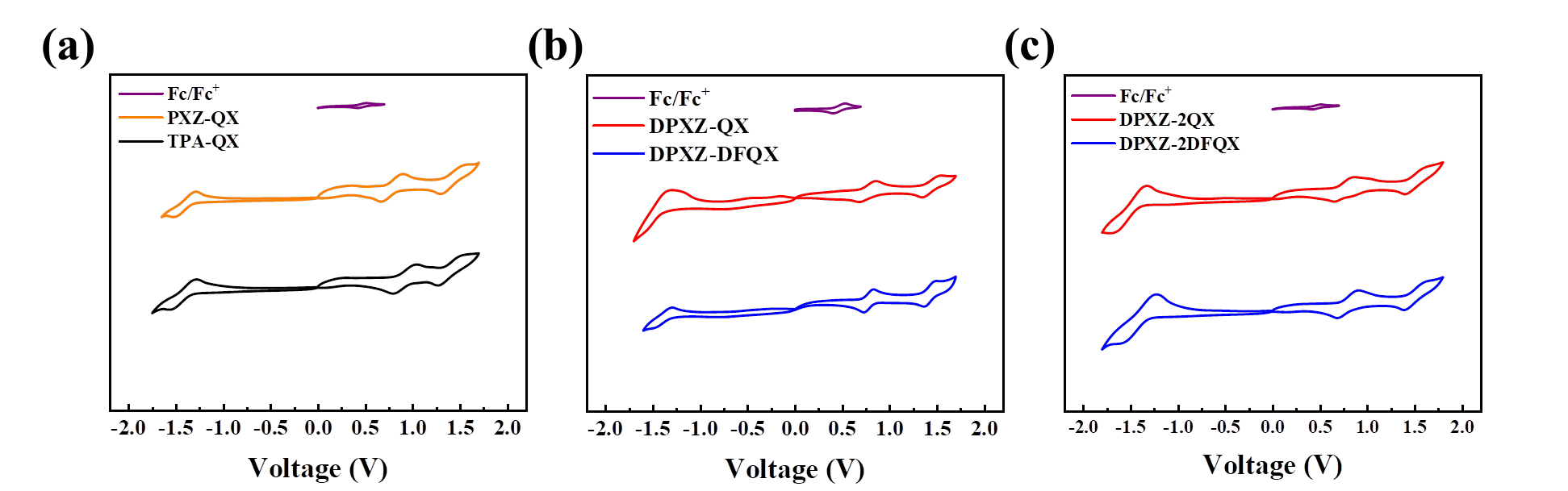
**

Figure S13. CV curves of PXZ-QX, TPA-QX, DPXZ-QX, DPXZ-DFQX, DPXZ-2QX and DPXZ-2DFQX in dichloromethane with an Ag/AgCl standard electrode as the reference electrode. The oxidation of ferrocene occurs at E_1/2_ = 0.47 V under the same condition.


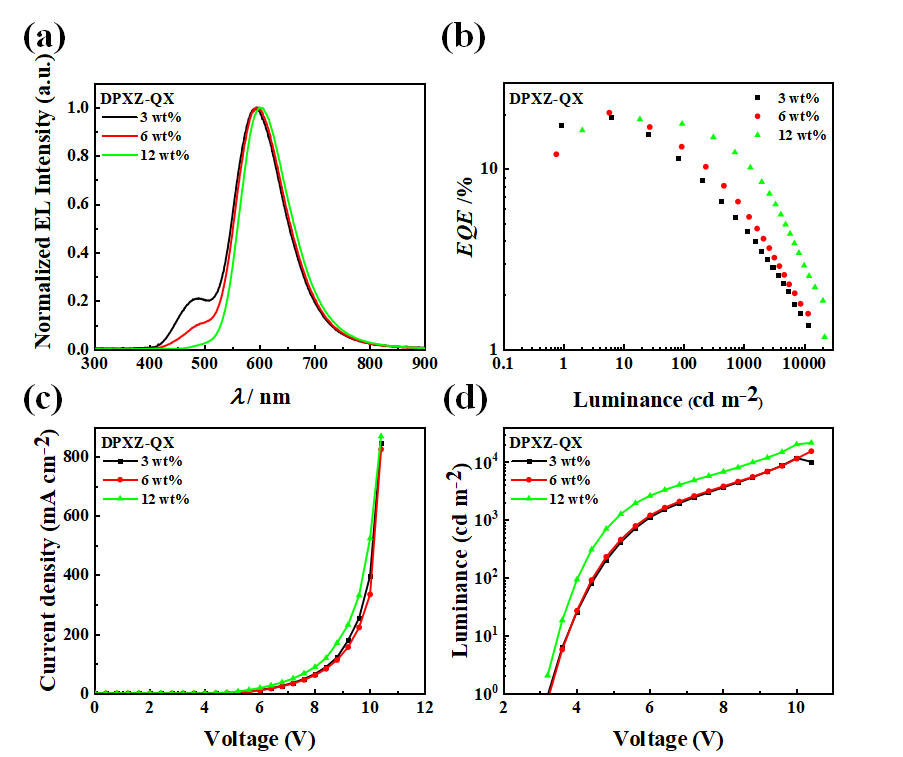


# Figure S14. Normalized EL spectra, EQE-luminance characteristics, and current density-luminance-voltage characteristics of OLEDs with DPXZ-QX-doped mCBP as the emitting layer.


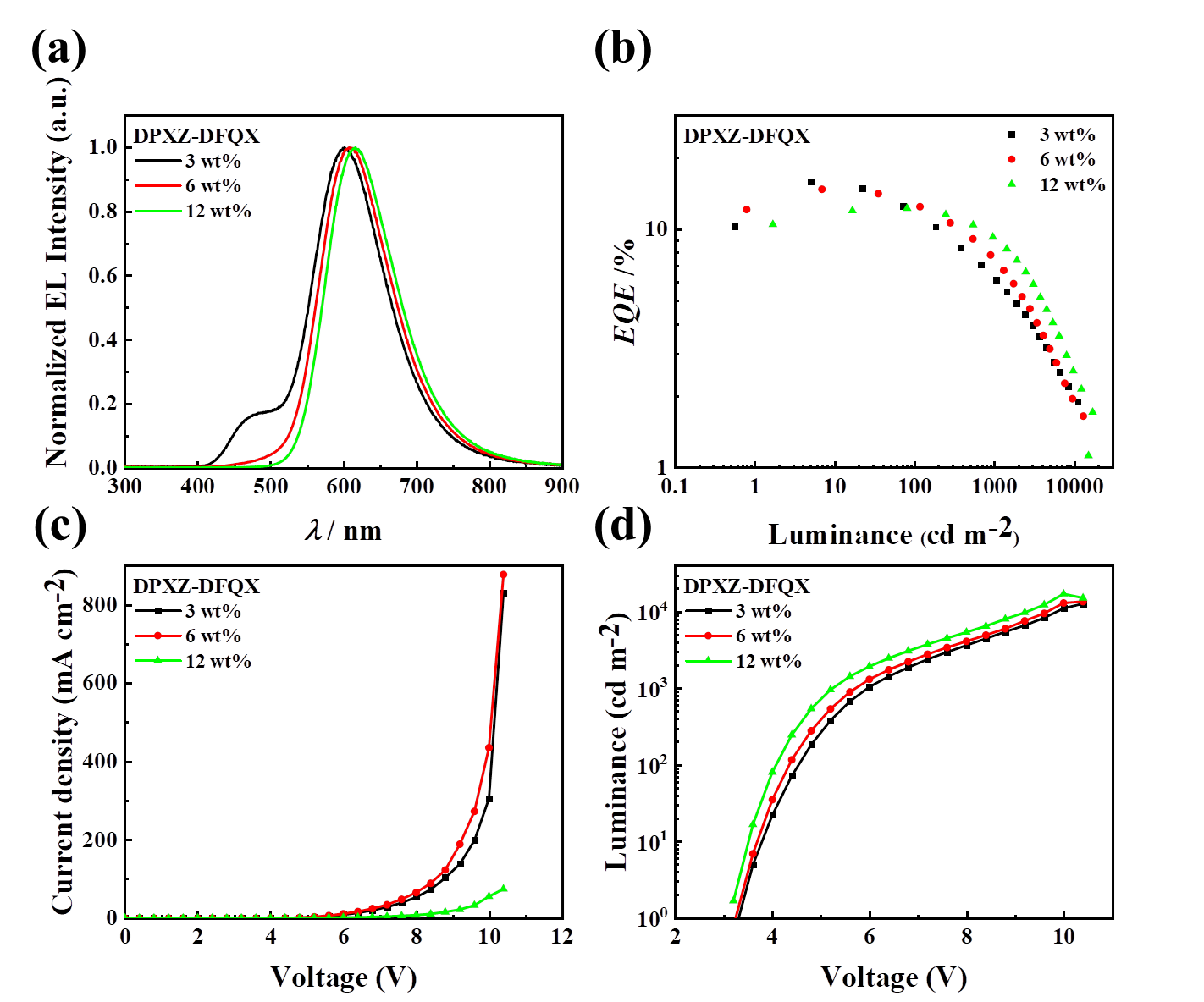


Figure S15. Normalized EL spectra, EQE-luminance characteristics, and current density-luminance-voltage characteristics of OLEDs with DPXZ-DFQX-doped mCBP as the emitting layer.


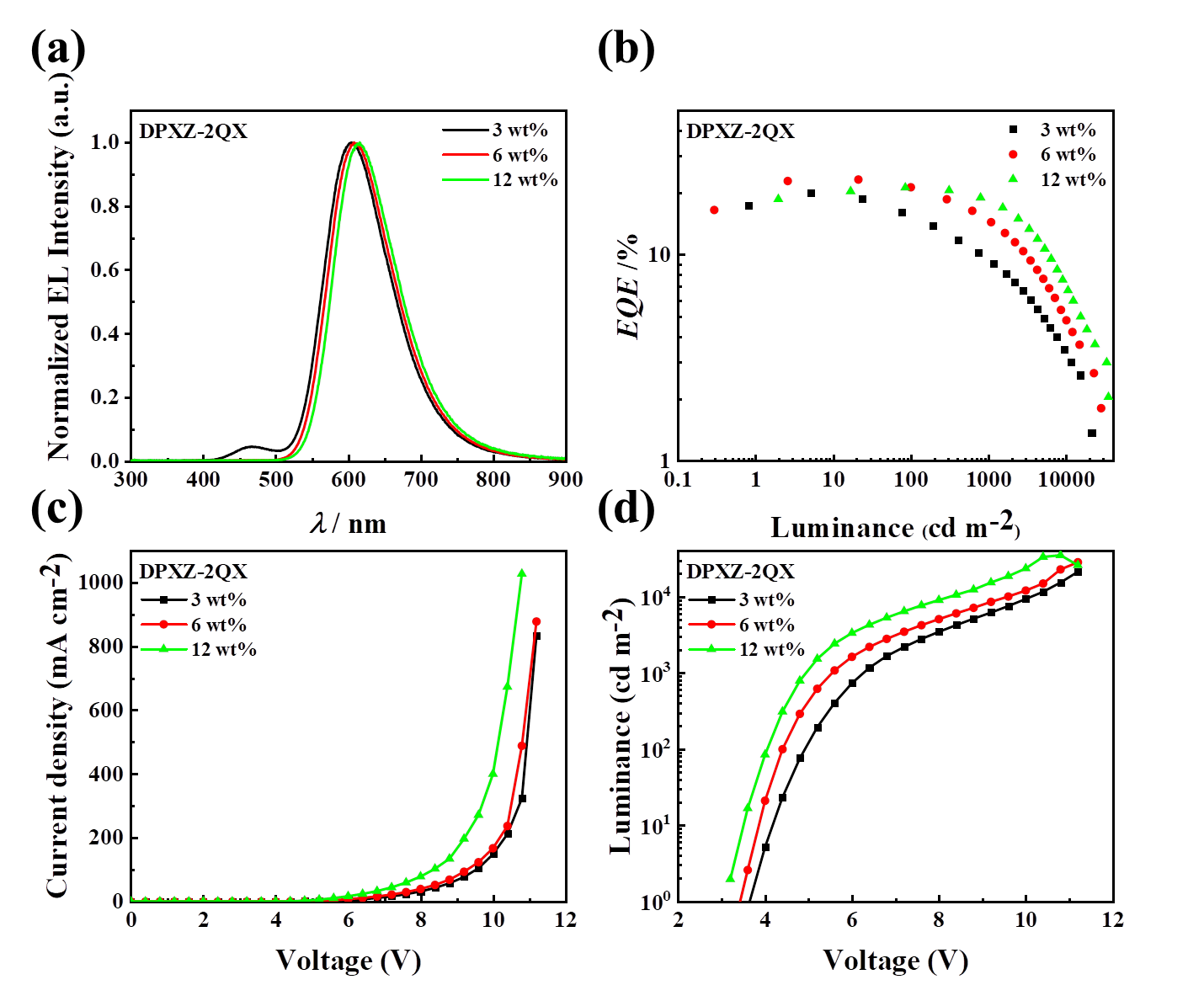


Figure S16. Normalized EL spectra, EQE-luminance characteristics, and current density-luminance-voltage characteristics of OLEDs with DPXZ-2QX-doped mCBP as the emitting layer.


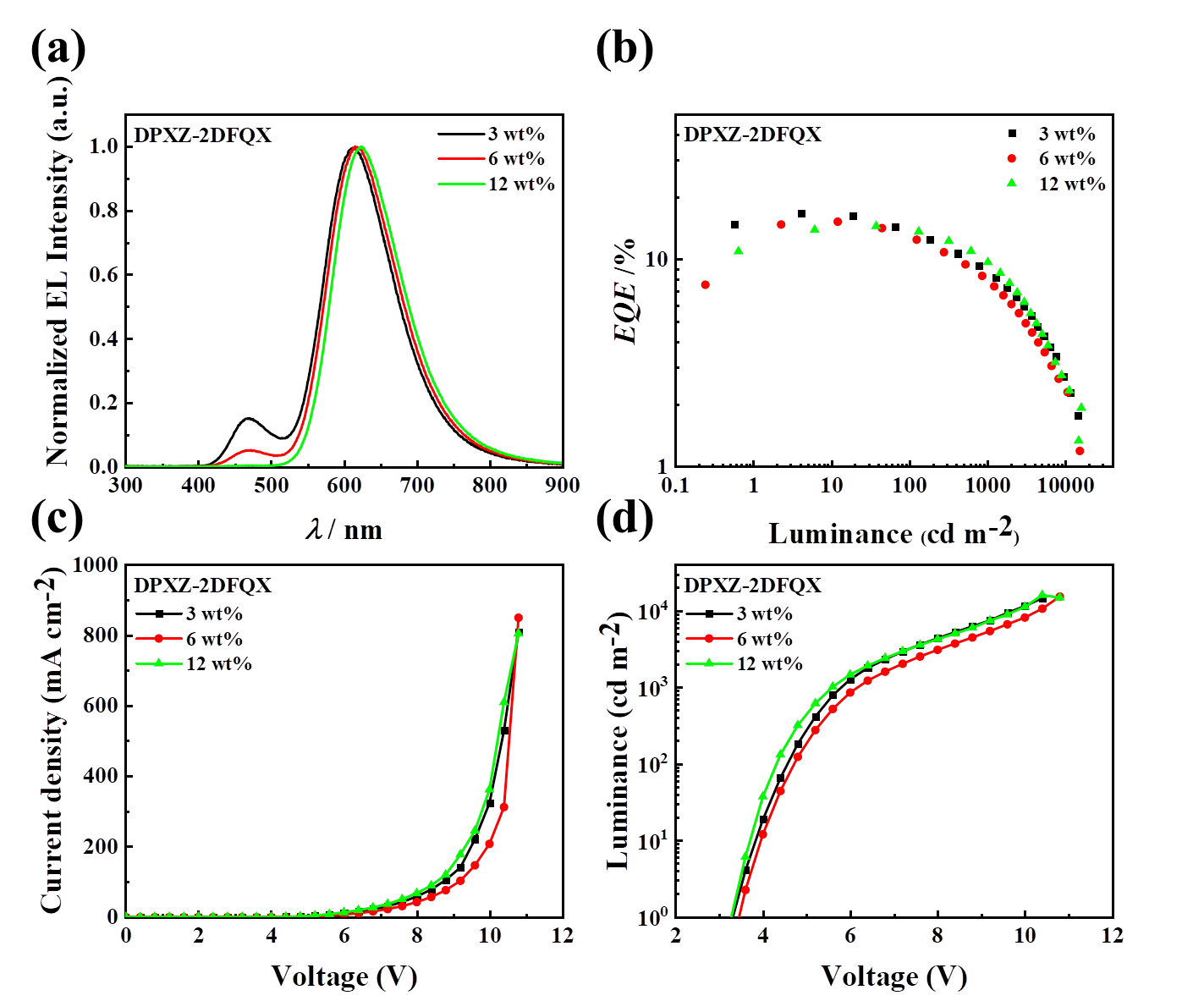


# Figure S17. Normalized EL spectra, EQE-luminance characteristics, and current density-luminance-voltage characteristics of OLEDs with DPXZ-2DFQX-doped mCBP as the emitting layer.

# Table S6. Summary of key device data based on DPXZ-DFQX and DPXZ-2DFQX.

| conc. | *L*  [cd m^-2^]^[a]^ | CE  [cd A^-1^]^[b]^ | PE  [lm W^-1^]^[b]^ | EQE  [%]^[b]^ | λ_max_  [nm]^[c]^ | CIE  [(x, y)]^[c]^ |
| --- | --- | --- | --- | --- | --- | --- |
| **DPXZ-DFQX** | | | | | | |
| 3 wt% | 12700 | 32.94; 11.95 | 28.75; 6.26 | 15.80; 6.10 | 602 | 0.50, 0.42 |
| 6 wt% | 13600 | 26.12; 13.63 | 22.79; 7.65 | 14.69; 7.79 | 609 | 0.56, 0.43 |
| 12 wt% | 15200 | 18.16; 13.92 | 15.28; 8.41 | 12.23; 9.27 | 617 | 0.59, 0.41 |
| **DPXZ-2DFQX** | | | | | | |
| 3 wt% | 14700 | 26.11; 14.83 | 22.79; 8.32 | 16.61; 9.28 | 616 | 0.53, 0.39 |
| 6 wt% | 15300 | 21.86; 12.17 | 17.17; 6.37 | 15.21; 8.33 | 618 | 0.58, 0.39 |
| 12 wt% | 16100 | 17.43; 12.15 | 13.69; 6.82 | 14.47; 9.69 | 625 | 0.61, 0.39 |

[a] Maximum luminance; [b] Values of current efficiency (CE), power efficiency (PE), and external quantum efficiency (EQE) at maximum and 1000 cd m^-2^; [c] λ_max_ and CIE coordinates at 1000 cd m^-2^.

# Table S7. Device data of the doped red TADF OLEDs (EL_max_ ≥ 590 nm) based on selected representative emitters in the literature and comparison with this work.

| Type | Emitter | EQE_max_  [%] | EQE_1000cd m-2_  [%] | λ_EL,max_  [nm] | Ref. |
| --- | --- | --- | --- | --- | --- |
| TSCT | DPXZ-2QX (6 wt%) | 23.2 | 14.4 | 609 | This work |
| TSCT | DPXZ-2QX (12 wt%) | 21.1 | 18.9 | 616 | This work |
| TSCT | RD-2TF | 10.3 | 7.8 | 626 | [9] |
| TSCT | P5-05 | 1.0 | 0.3 | 616 | [10] |
| TBCT | DPXZ-BPPZ | 20.1 | 16.7 | 612 | [11] |
| TBCT | BPPZ-PXZ | 25.2 | 18.1 | 604 | [12] |
| TBCT | mDPBPZ-PXZ | 21.7 | 14.6 | 624 |  |
| TBCT | 3DMAc-BP | 22.0 | 9.7 | 606 | [13] |
| TBCT | DBPZ-DPXZ | 17.8 | 13.0 | 608 | [14] |
|  | tDBBPZ-DPXZ | 17.2 | 8.9 | 608 |  |
| TBCT | oDTBPZ-DPXZ | 20.1 | < 9.5 | 604 | [15] |
| TBCT | FBPCNAc | 17.4 | - | 614 | [16] |
| TBCT | PY-TPA | 20.09 | - | 639 | [17] |
|  | CT3-TPA | 18.81 | - | 638 |  |
|  | CN-TPA | 20.1 | - | 676 |  |
| TBCT | PQ2 | 17.3 | 14.7 | 605 | [18] |
| TBCT | DCPPr-α-NDPA | 31.5 | - | 606 | [19] |
| TBCT | NAI-R3 | 22.5 | 3.4 | 622 | [20] |
| TBCT | mCPCN | 31.7 | 6.4 | 593 | [21] |
| TBCT | dPhADBA | 11.1 | 8.8 | 613 | [22] |
| TBCT | PzDBA | 21.8 | 20.1 | 595 | [23] |
| TBCT | 4tBuMB | 19.4 | >17.2 | 617 | [24] |
| TBCT | PT-TPA | 29.7 | - | 632 | [25] |

# References

[1] Q. Hao, S. Yu, S. Li, J. Chen, Y. Zeng, T. Yu, G. Yang, Y. Li, J. Org. Chem. 2014, 79, 459-464.

[2] J. Klajn, W. Stawski, PJ. Chmielewski, J. Cybinska, M. Pawlicki. Chem. Commun. 2019, 55, 4558-4561.

[3] O. V. Dolomanov, L. J. Bourhis, R. J. Gildea, J. A. K. Howard, H. Puschmann, *J. Appl. Cryst.* **2009**, *42*, 339-341.

[4] G. M. Sheldrick, *Acta. Cryst.* **2015**, *A71*, 3-8.

[5] G. M. Sheldrick, *Acta. Cryst.* **2015**, *C71*, 3-8.

[6] M. J. Frisch, G. W. Trucks, H. B. Schlegel, G. E. Scuseria, M. A. Robb, J. R. Cheeseman, G. Scalmani, V. Barone, B. Mennucci, G. A. Petersson, H. Nakatsuji, M. Caricato, X. Li, H. P. Hratchian, A. F. Izmaylov, J. Bloino, G. Zheng, J. L. Sonnenberg, M. Hada, M. Ehara, K. Toyota, R. Fukuda, J. Hasegawa, M. Ishida, T. Nakajima, Y. Honda, O. Kitao, H. Nakai, T. Vreven, J. A. Montgomery Jr, J. E. Peralta, F. Ogliaro, M. Bearpark, J. J. Heyd, E. Brothers, K. N. Kudin, V. N. Staroverov, R. Kobayashi, J. Normand, K. Raghavachari, A. Rendell, J. C. Burant, S. S. Iyengar, J. Tomasi, M. Cossi, N. Rega, J. M. Millam, M. Klene, J. E. Knox, J. B. Cross, V. Bakken, C. Adamo, J. Jaramillo, R. Gomperts, R. E. Stratmann, O. Yazyev, A. J. Austin, R. Cammi, C. Pomelli, J. W. Ochterski, R. L. Martin, K. Morokuma, V. G. Zakrzewski, G. A. Voth, P. Salvador, J. J. Dannenberg, S. Dapprich, A. D. Daniels, Ö . Farkas, J. B. Foresman, J. V. Ortiz, J. Cioslowski, D. J. Fox, Revision D.01 ed., Gaussian, Inc., Wallingford CT, **2009**.

[7] M. J. Frisch, G. W. Trucks, H. B. Schlegel, G. E. Scuseria, M. A. Robb, J. R. Cheeseman, G. Scalmani, V. Barone, B. Mennucci and G. A. Petersson, *Theor. Chem. Acc.*, **2008**, *120*, 215.

[8] R. L. Martin, *J. Chem. Phys.*, **2003**, *118*, 4775-4777.

[9] X. Wang, J. Hu, J. Lv, Q. Yang, H. Tian, S. Shao, L. Wang, X. Jing, F. Wang, *Angew. Chem. Int. Ed.* **2021**, *60*, 16585-16593.

[10] J. Hu, Q. Li, X. Wang, S. Shao, L. Wang, X. Jing, F. Wang, *Angew. Chem. Int. Ed.* **2019**, *58*, 8405-8409.

[11] J. X. Chen, K. Wang, C. J. Zheng, M. Zhang, Y. Z. Shi, S. L. Tao, H. Lin, W. Liu, W. W. Tao, X. M. Ou, X. H. Zhang, Adv. Sci. 2018, 5, 1800436.

[12] J. X. Chen, W. W. Tao, W. C. Chen, Y. F. Xiao, K. Wang, C. Cao, J. Yu, S. Li, F. X. Geng, C. Adachi, C. S. Lee, X. H. Zhang, Angew. Chem., Int. Ed. 2019, 58, 14660-14665.

[13] F. M. Xie, H. Z. Li, G. L. Dai, Y. Q. Li, T. Cheng, M. Xie, J. X. Tang, X. Zhao, ACS Appl. Mater. Interfaces 2019, 11, 26144-26151.

[14] J. X. Chen, W. W. Tao, Y. F. Xiao, K. Wang, M. Zhang, X. C. Fan, W. C. Chen, J. Yu, S. Li, F. X. Geng, X. H. Zhang, C. S. Lee, ACS Appl. Mater. Interfaces 2019, 11, 29086-29093.

[15] J. X. Chen, Y. F. Xiao, K. Wang, D. Sun, X. C. Fan, X. Zhang, M. Zhang, Y. Z. Shi, J. Yu, F. X. Geng, C. S. Lee, X. H. Zhang, Angew. Chem., Int. Ed. 2021, 60, 2478-2484.

[16] S. Kothavale, W. J. Chung, J. Y. Lee, ACS Appl. Mater. Interfaces 2020, 12, 18730-18738.

[17] J. L. He, F. C. Kong, B. Sun, X.-J. Wang, Q. S. Tian, J. Fan, L. S. Liao, Chem. Eng. J. 2021, 424, 130470.

[18] U. Balijapalli, Y. T. Lee, B. S. B. Karunathilaka, G. Tumen-Ulzii, M. Auffray, Y. Tsuchiya, H. Nakanotani, C. Adachi, *Angew. Chem., Int. Ed.* 2021, 60, 19364-19373.

[19] Z. Cai, X. Wu, H. Liu, J. Guo, D. Yang, D. Ma, Z. Zhao, B. Z. Tang, *Angew. Chem., Int. Ed.* 2021, 60, 23635-23640.

[20] W. Zeng, T. Zhou, W. Ning, C. Zhong, J. He, S. Gong, G. Xie, C. Yang, Adv. Mater. 2019,

31, 1901404.

[21] X. Zeng, Y. H. Huang, S. Gong, P. Li, W. K. Lee, X. Xiao, Y. Zhang, C. Zhong, C. C. Wu,

C. Yang, Mater. Horiz. 2021, 8, 2286-2292.

[22] C. M. Hsieh, T. L. Wu, J. Jayakumar, Y. C. Wang, C. L. Ko, W. Y. Hung, T. C. Lin, H. H.

Wu, K. H. Lin, C. H. Lin, S. Hsieh, C. H. Cheng, ACS Appl. Mater. Interfaces 2020, 12, 23199-

23206.

[23] D. Karthik, Y. H. Jung, H. Lee, S. Hwang, B. M. Seo, J. Y. Kim, C. W. Han, J. H. Kwon,

Adv. Mater. 2021, 33, 2007724.

[24] Y. H. Jung, D. Karthik, H. Lee, J. H. Maeng, K. J. Yang, S. Hwang, J. H. Kwon, ACS Appl.

Mater. Interfaces 2021, 13, 17882-17891.

[25] Y.Y. Wang, K.N. Tong, K. Zhang, C.H. Lu, X. Chen, J.X. Liang, C.K. Wang, C.C. Wu, M.K. Fung, J. Fan, Mater. Horiz. 2021, 8, 1297–1303.

# NMR Spectra


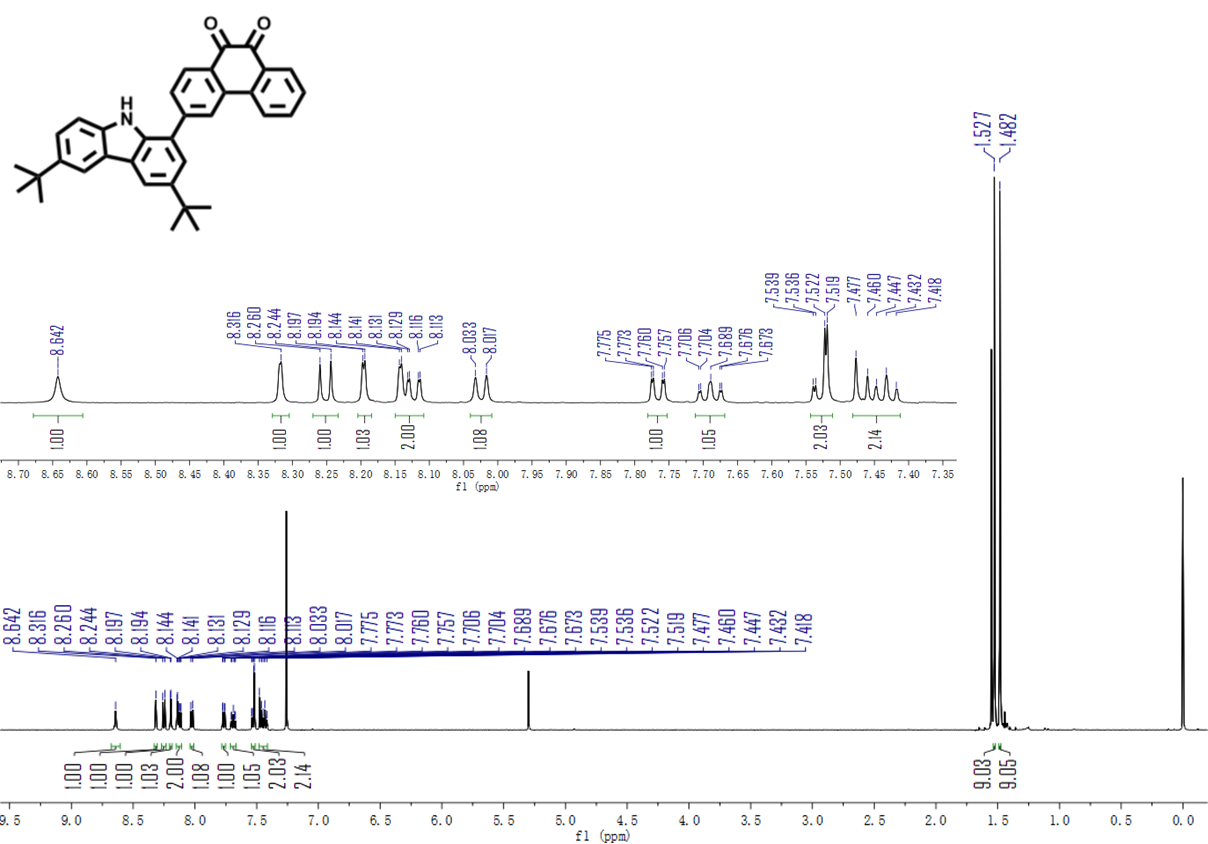


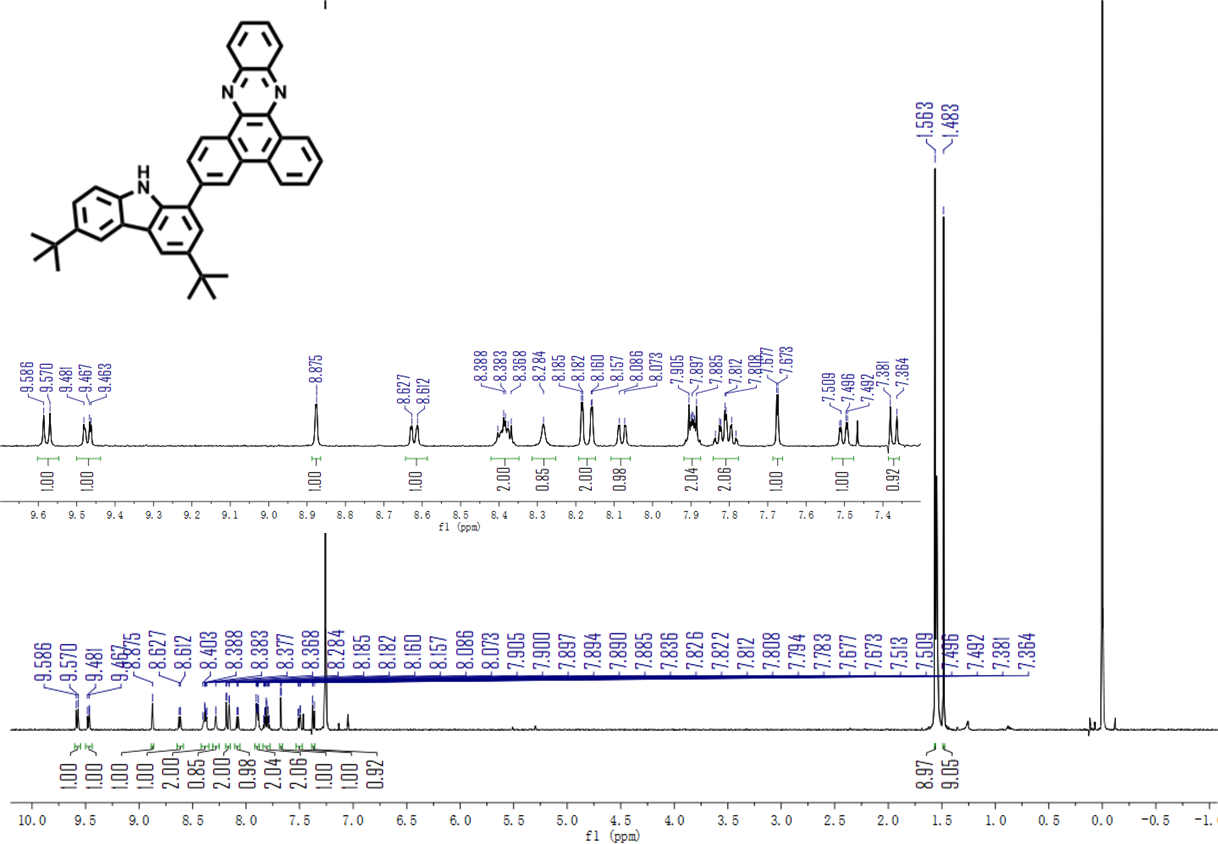


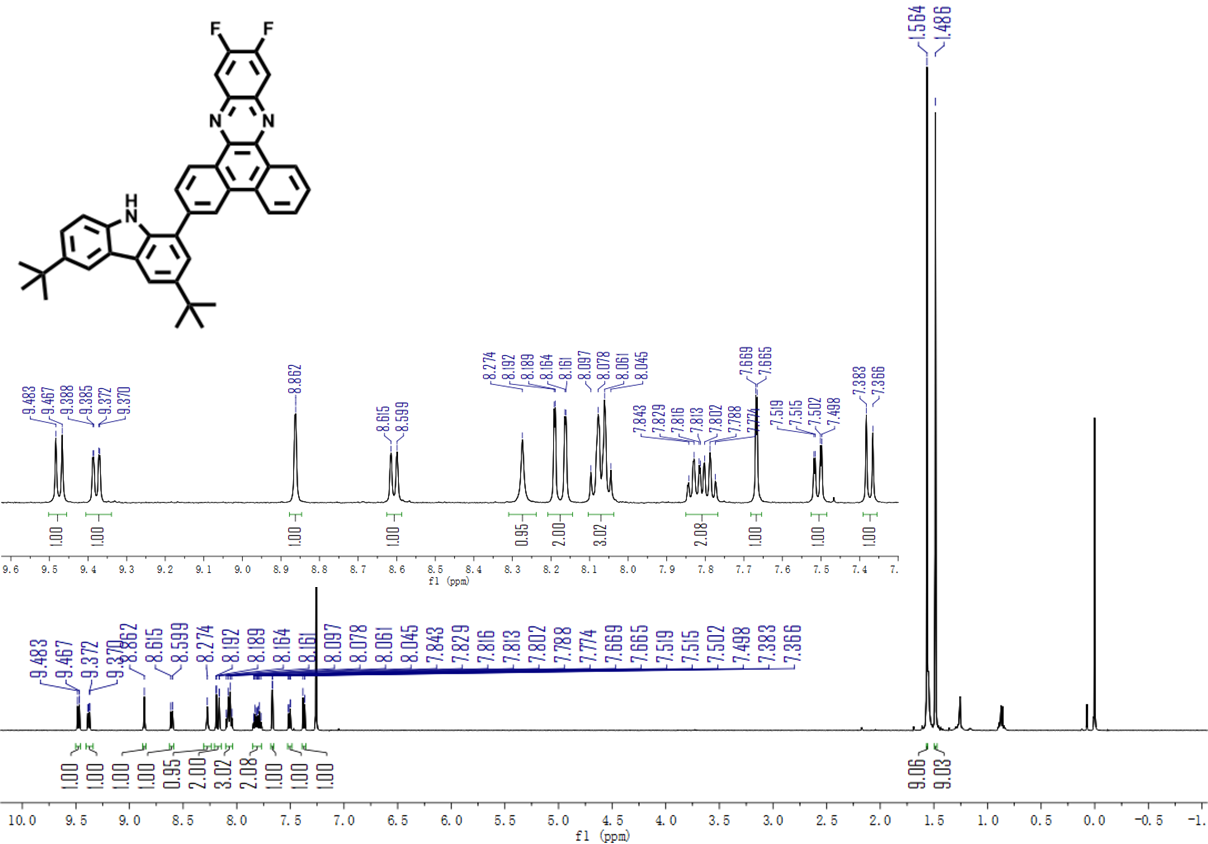


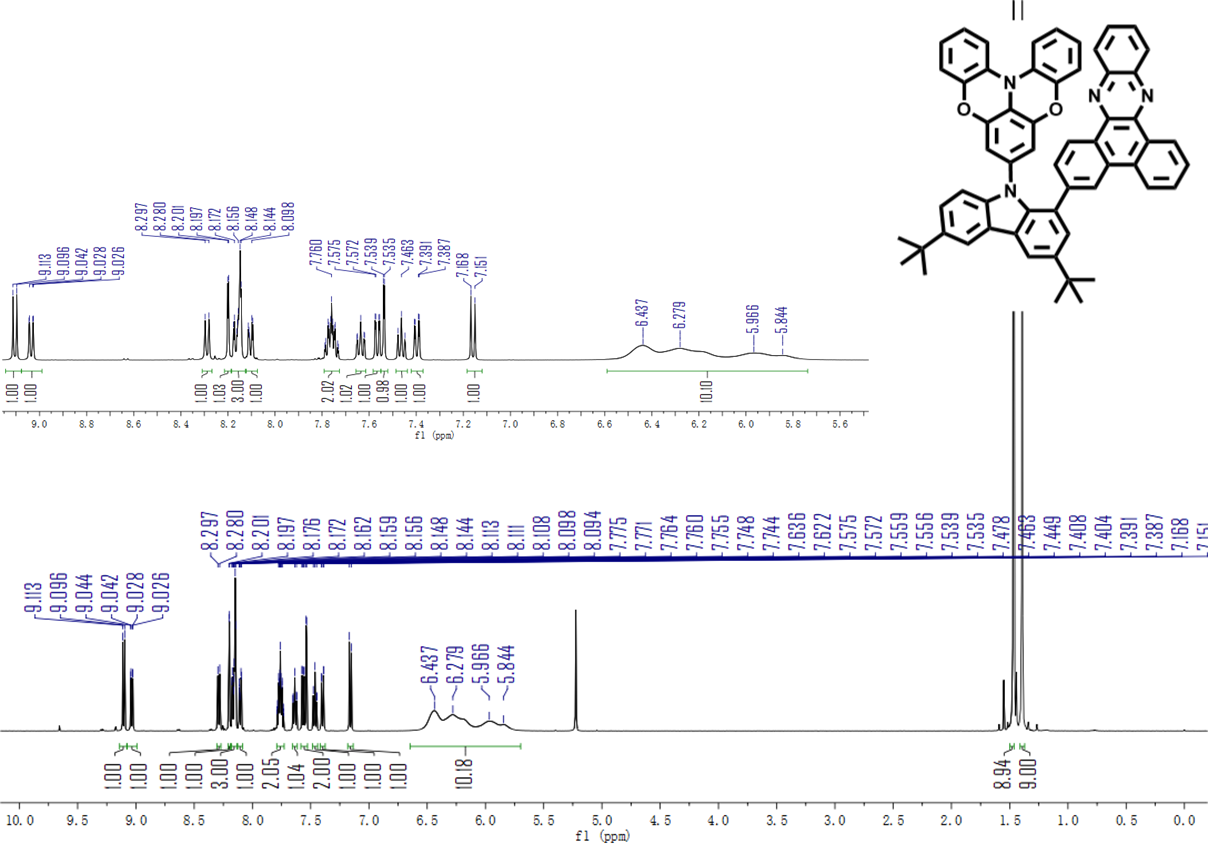


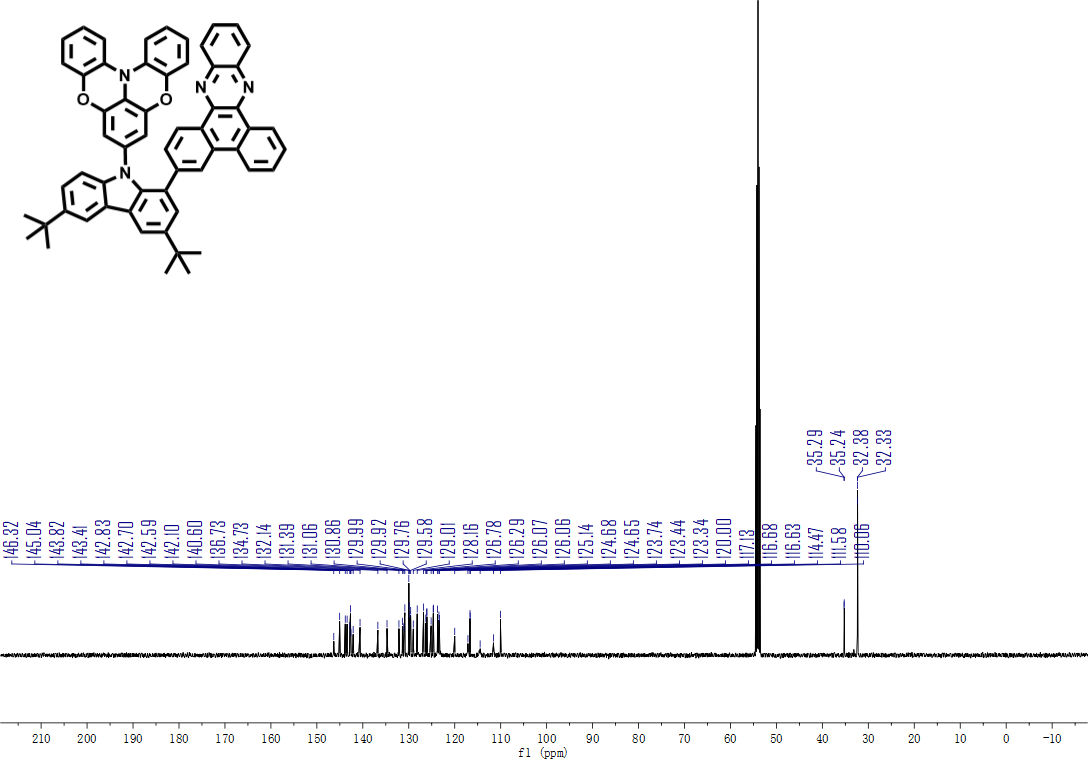


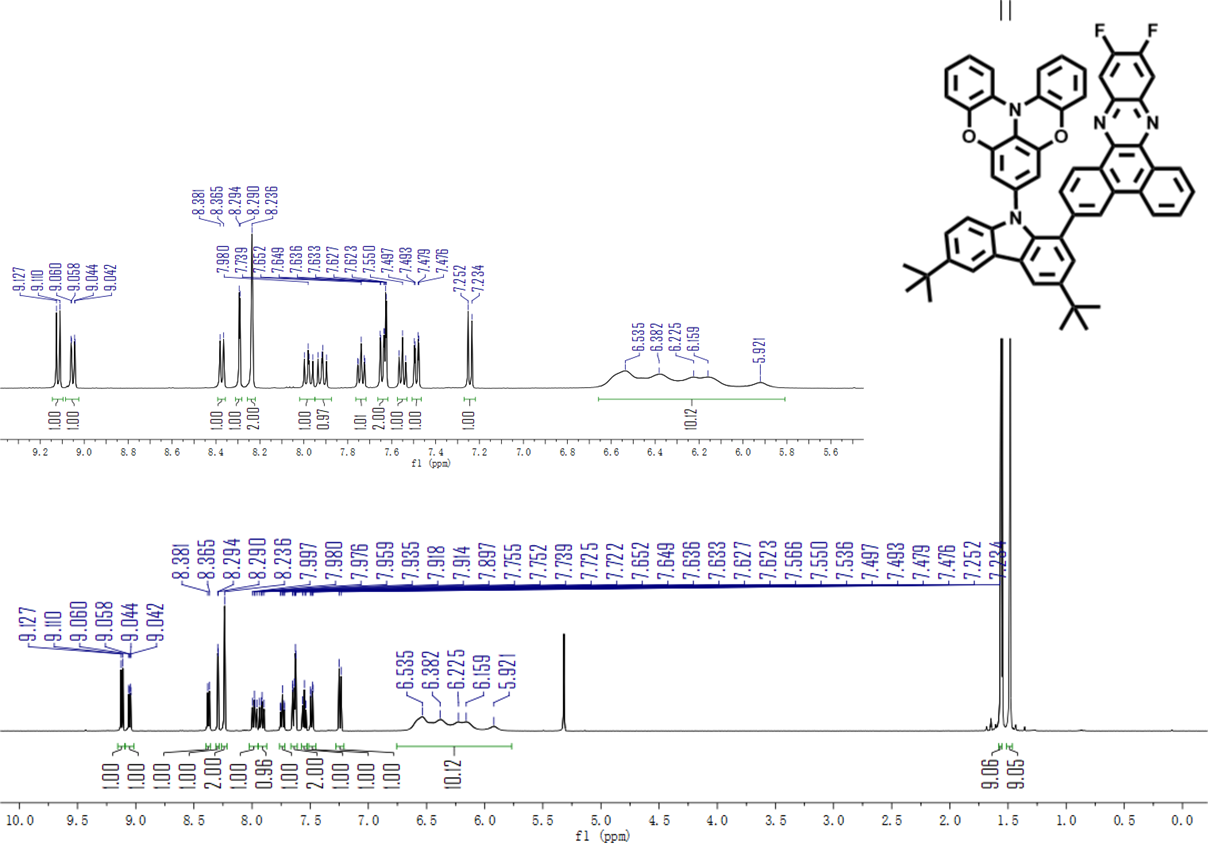


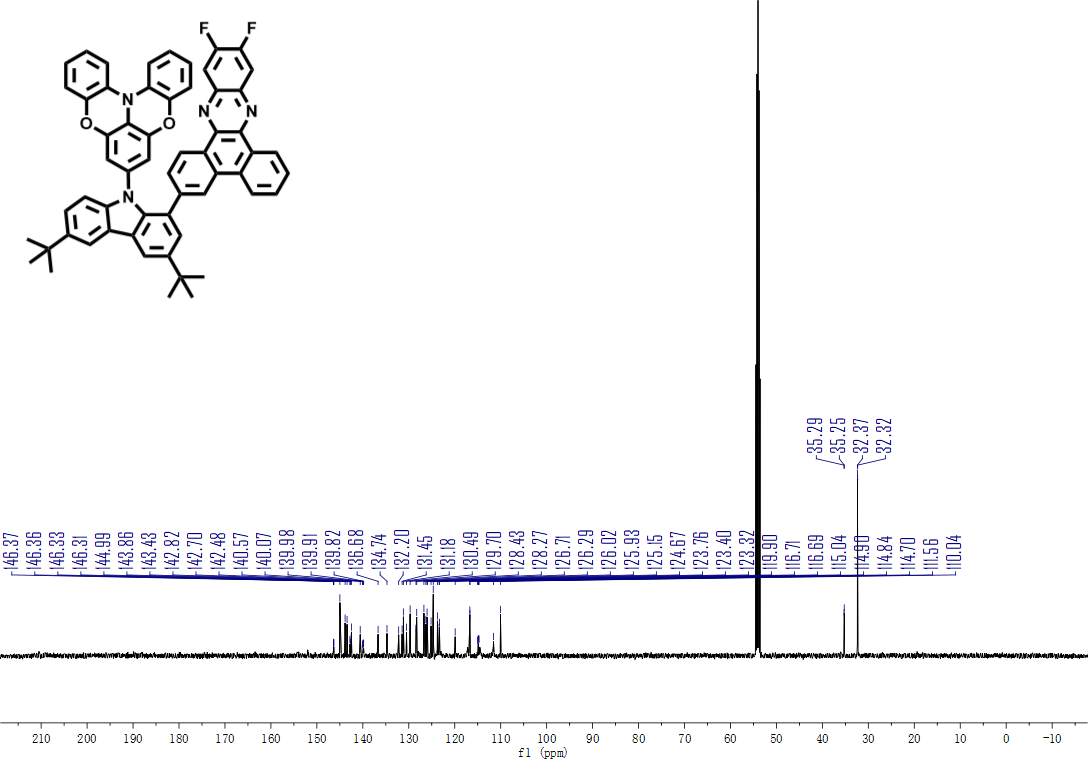


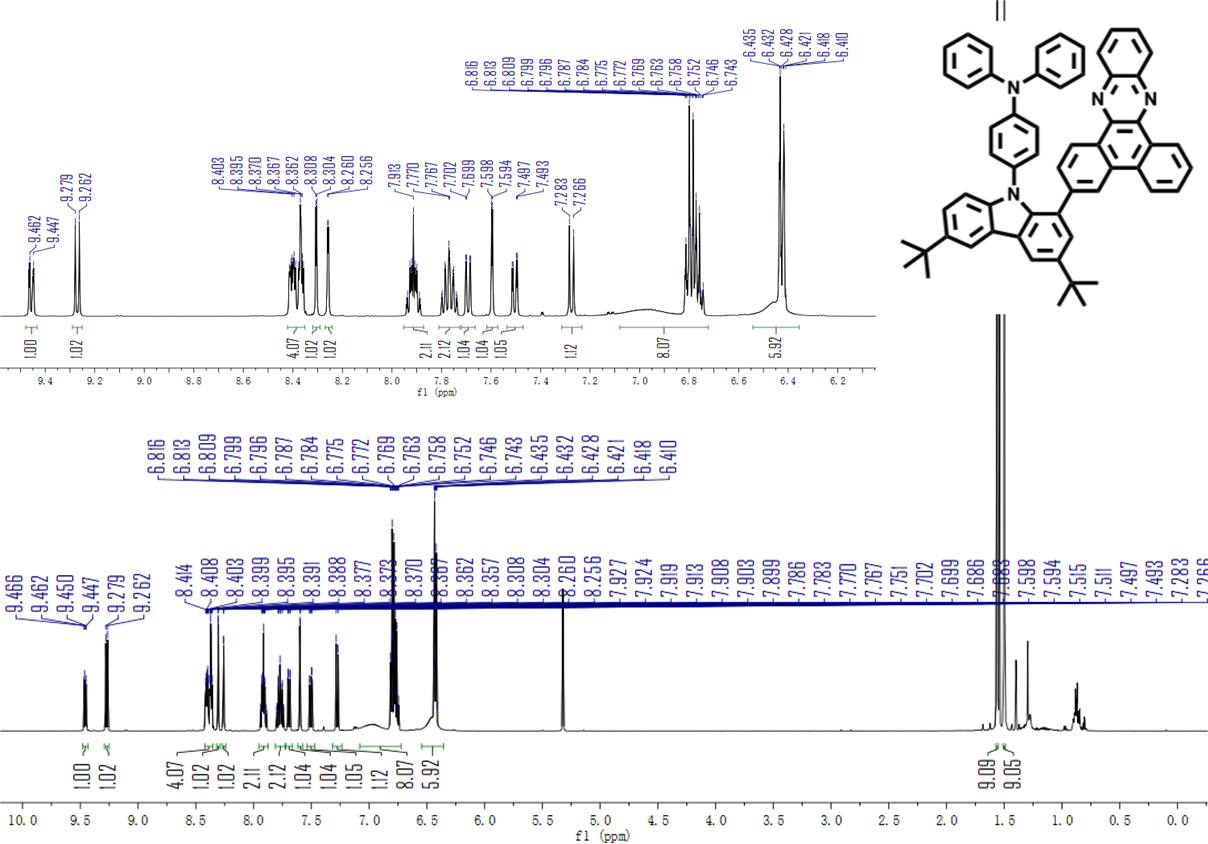


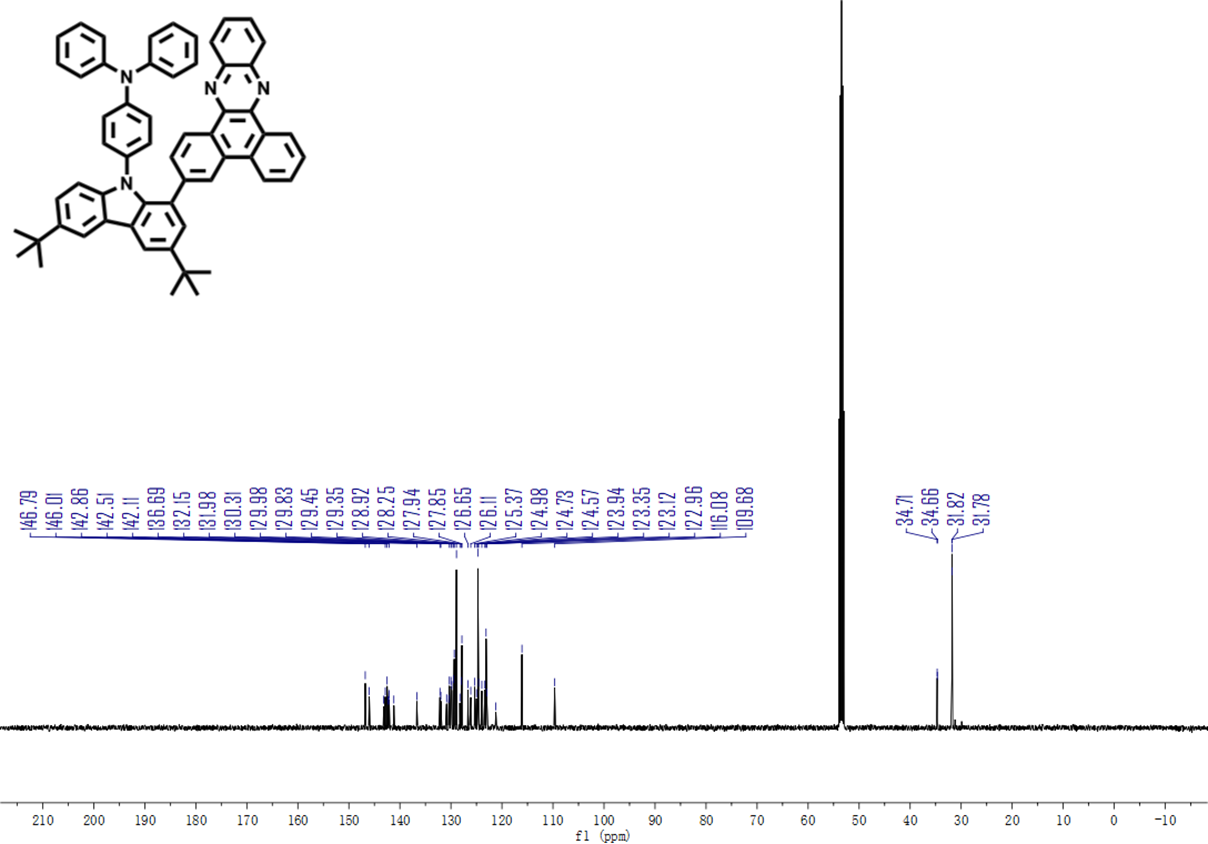


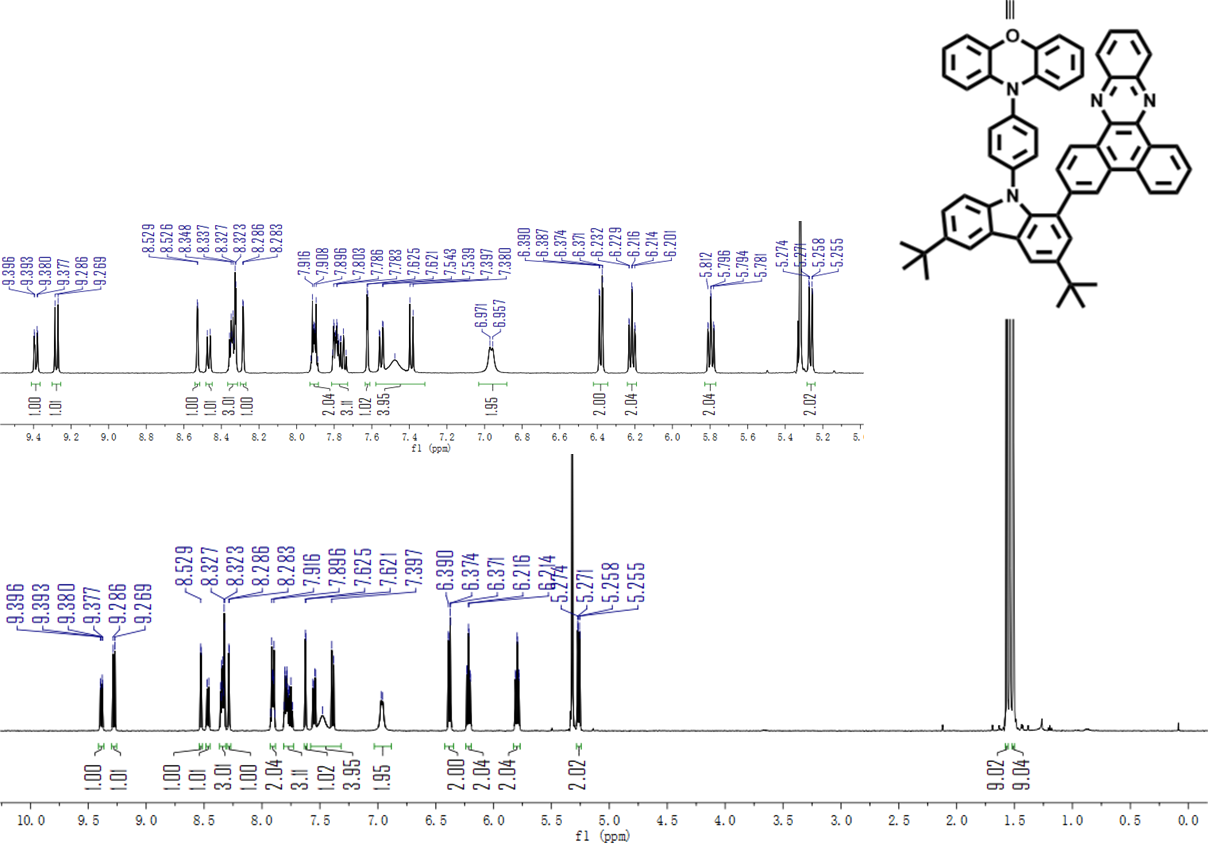


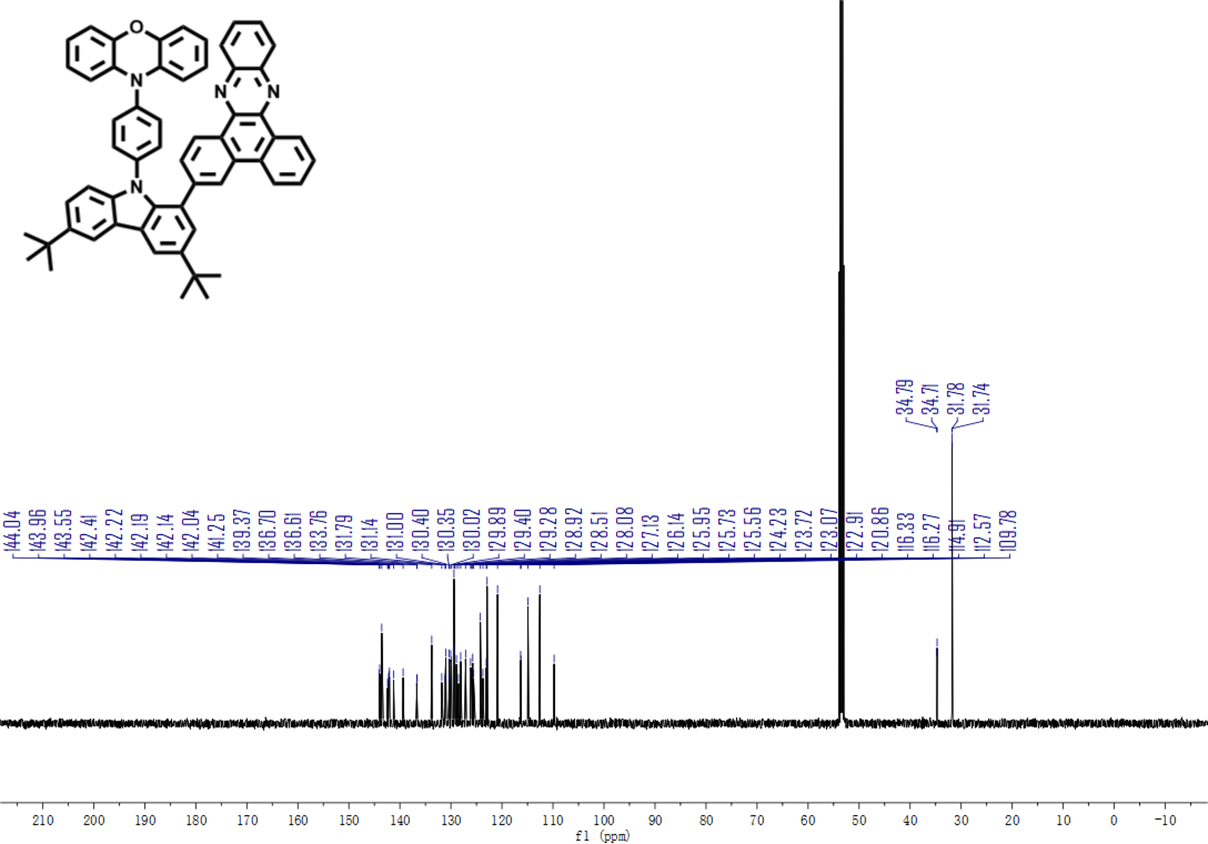


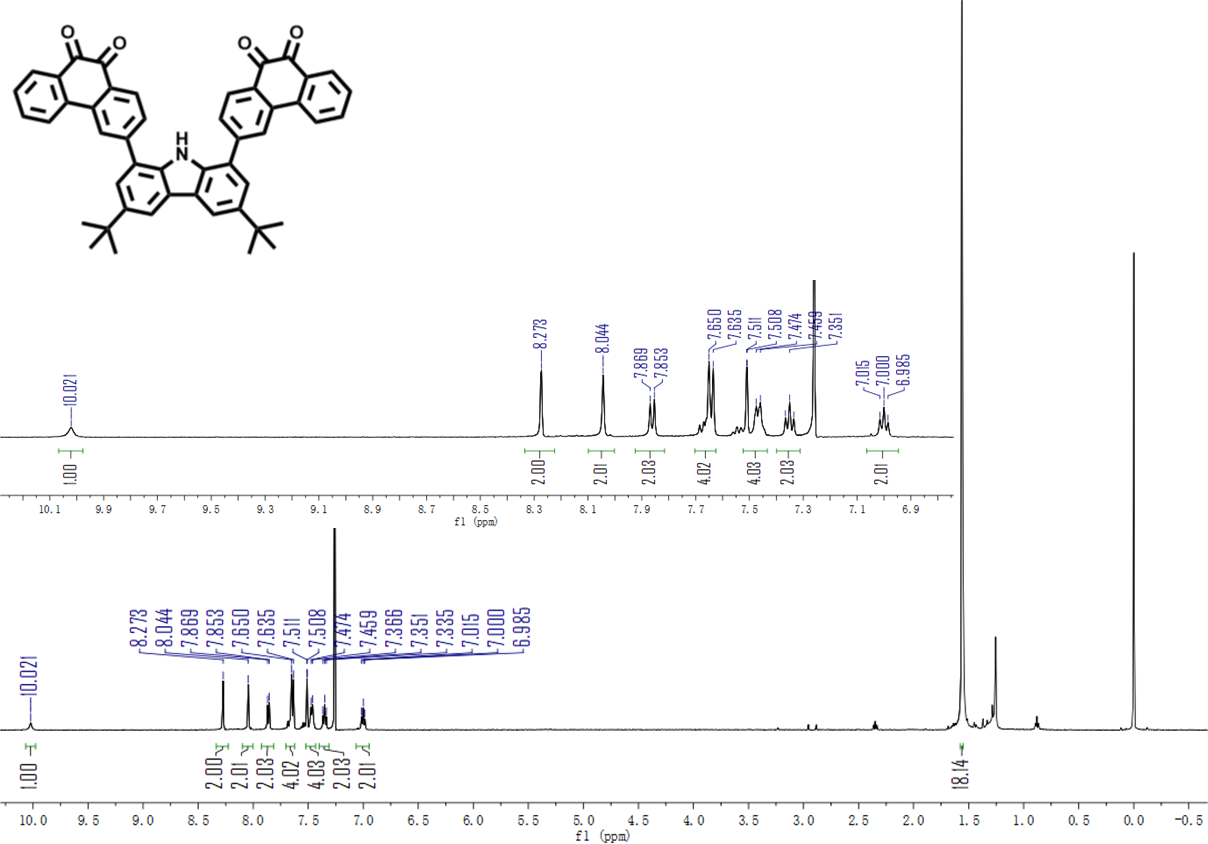


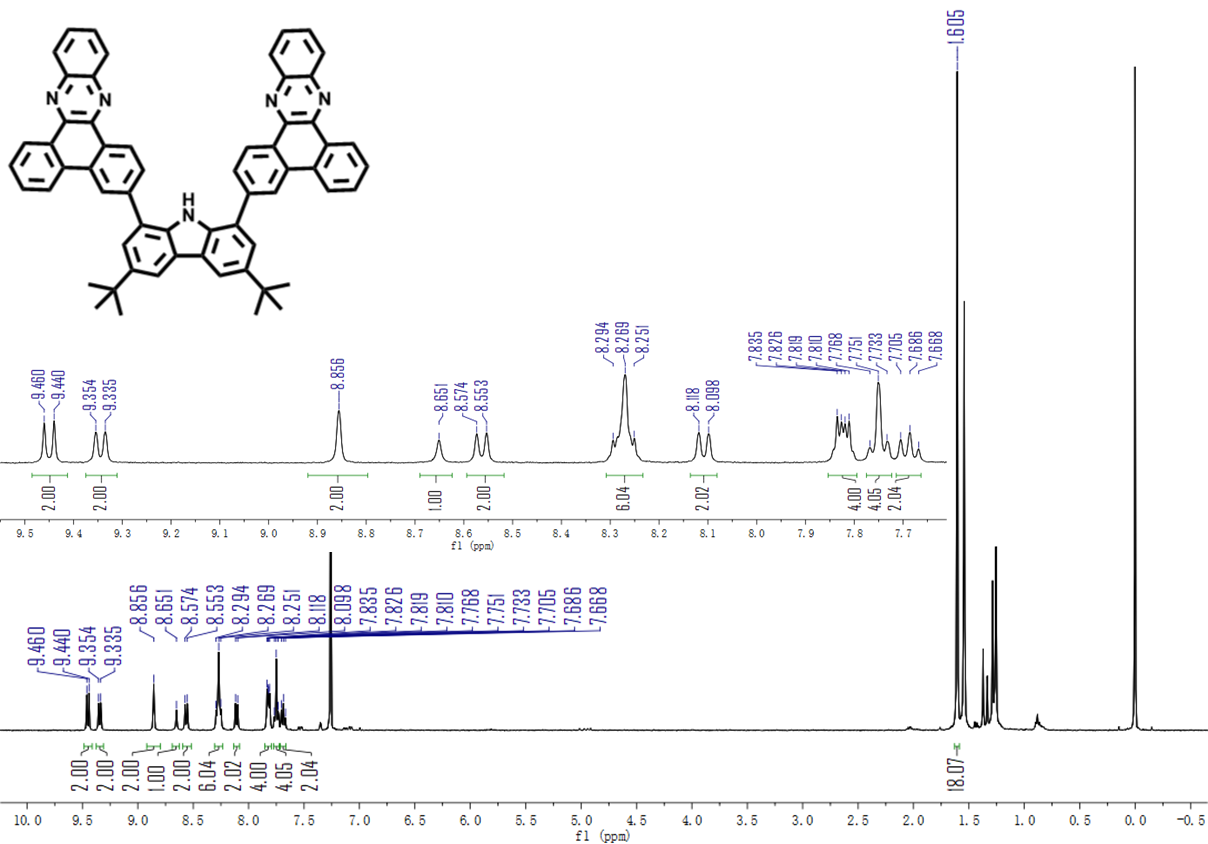


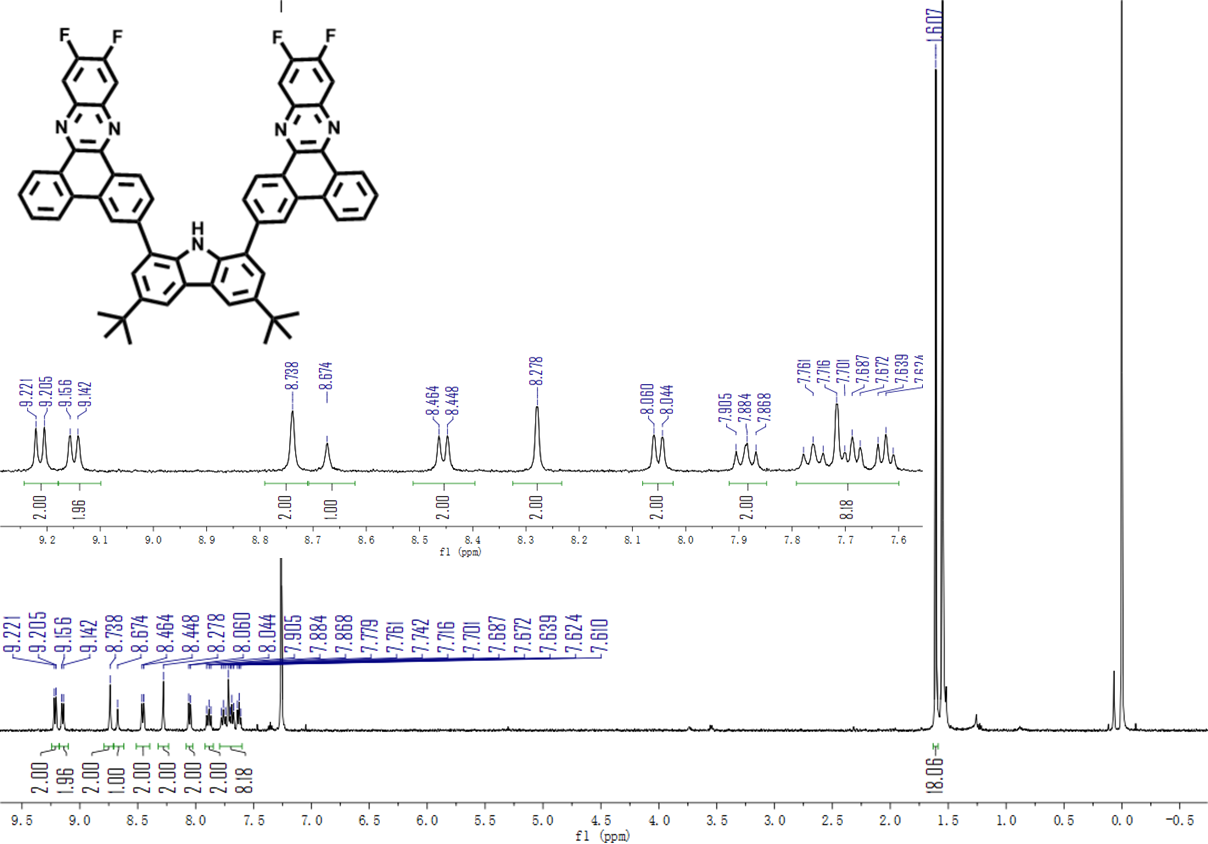


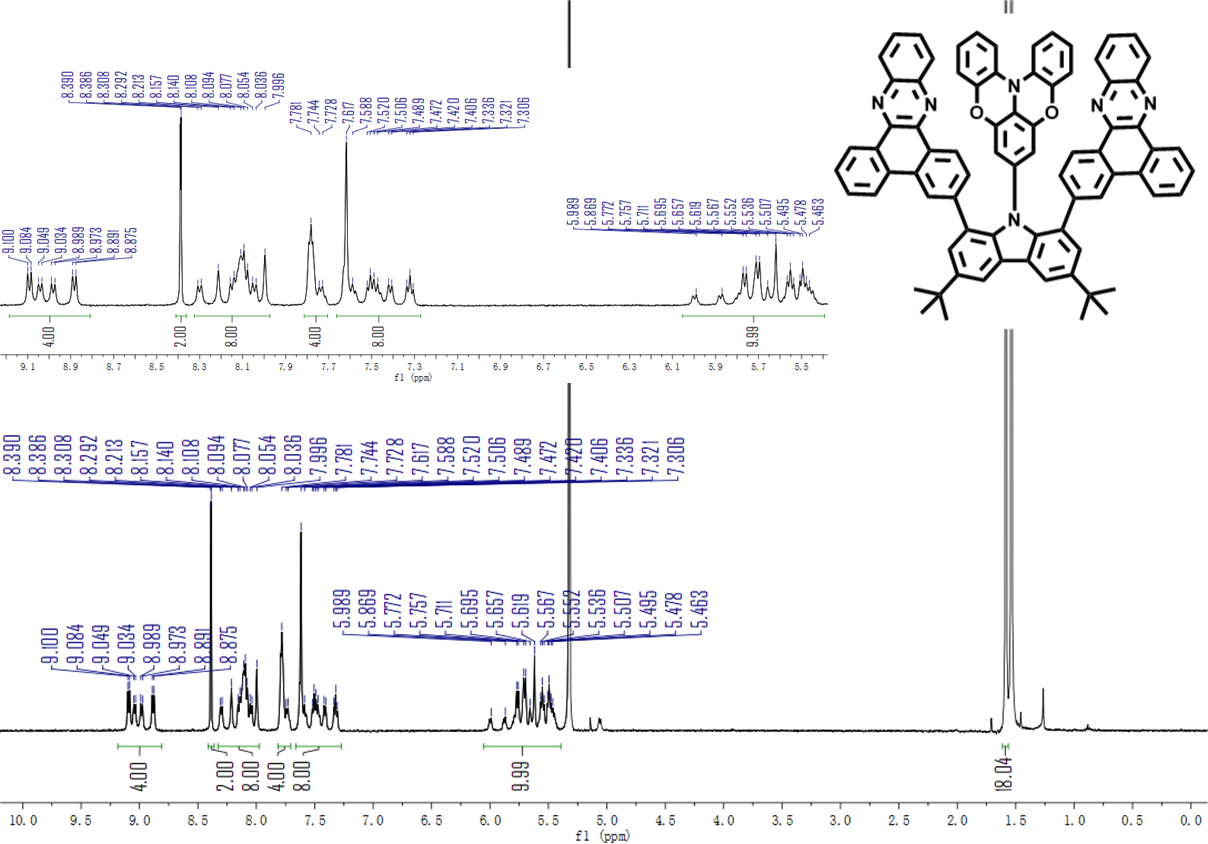


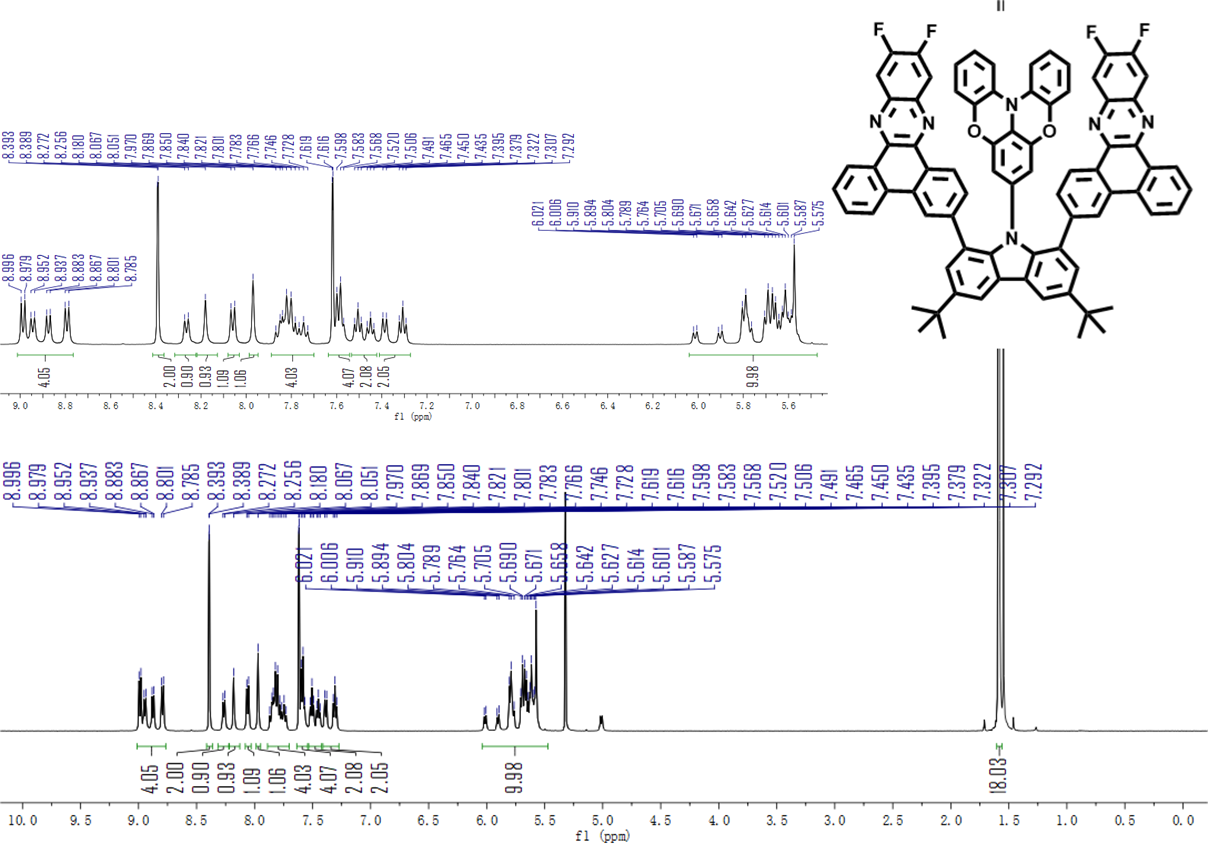

Supplement: Supplementary Materials — Experiments including general materials and instrumentations. Methods of single crystal analysis, theoretical simulations, device fabrication, and measurement. Syntheses and characterizations of new compounds. Figure S1: illustration of TSCT molecule design and list of selected TSCT molecules in the literature report. Figure S2: thermal properties. Tables S1-S3: crystal data. Figure S3: crystal structures. Figure S4: noncovalent interaction analysis using RDG. Figure S5: analysis of electronic structures using DFT and TDDFT calculations. Figures S6-S12 and Tables S4-S5: photophysical properties. Figure S13: cyclic voltammograms. Figures S14-S17 and Table S6: OLED data. Table S7: comparison of the present device performances with those in the literature reports. [file 9892802.f1.docx]
